# Genome-wide analysis and expression profiles of *PdeMYB* transcription factors in colored-leaf poplar (*Populus deltoids*)

**DOI:** 10.1186/s12870-021-03212-1

**Published:** 2021-09-23

**Authors:** Weibing Zhuang, Xiaochun Shu, Xinya Lu, Tao Wang, Fengjiao Zhang, Ning Wang, Zhong Wang

**Affiliations:** Jiangsu Key Laboratory for the Research and Utilization of Plant Resources, Institute of Botany, Jiangsu Province and Chinese Academy of Sciences (Nanjing Botanical Garden Mem. Sun Yat-Sen), Nanjing, 210014 China

**Keywords:** Genome-wide, *PdeMYB* transcription factors, *Populus deltoids*, Expression profiles, Anthocyanin biosynthesis

## Abstract

**Background:**

*MYB* transcription factors, comprising one of the largest transcription factor families in plants, play many roles in secondary metabolism, especially in anthocyanin biosynthesis. However, the functions of the *PdeMYB* transcription factor in colored-leaf poplar remain elusive.

**Results:**

In the present study, genome-wide characterization of the *PdeMYB* genes in colored-leaf poplar (*Populus deltoids*) was conducted. A total of 302 *PdeMYB* transcription factors were identified, including 183 R2R3-MYB, five R1R2R3-MYB, one 4R-MYB, and 113 1R-MYB transcription factor genes. Genomic localization and paralogs of *PdeMYB* genes mapped 289 genes on 19 chromosomes, with collinearity relationships among genes. The conserved domain, gene structure, and evolutionary relationships of the *PdeMYB* genes were also established and analyzed. The expression levels of *PdeMYB* genes were obtained from previous data in green leaf poplar (L2025) and colored leaf poplar (QHP) as well as our own qRT-PCR analysis data in green leaf poplar (L2025) and colored leaf poplar (CHP), which provide valuable clues for further functional characterization of *PdeMYB* genes.

**Conclusions:**

The above results provide not only comprehensive insights into the structure and functions of *PdeMYB* genes but also provide candidate genes for the future improvement of leaf colorization in *Populus deltoids*.

**Supplementary Information:**

The online version contains supplementary material available at 10.1186/s12870-021-03212-1.

## Background

Transcription factors play important roles in the regulation of gene expression, which can control the rate of transcription initiation of target genes. *MYB* transcription factors, one of the largest and best-characterized transcription factor gene families in plants, are widely distributed in all eukaryotic organisms, including animals, plants, and fungi [1, 2], which can be involved in various types of plant growth and development.

Most transcription factors are structurally classified into different families based on their domain diversity [3, 4]. Similarly, *MYB* transcription factors can be classified into four types according to the number of MYB domains: 1R-MYB, R2R3-MYB, R1R2R3-MYB, and 4R-MYB [5–8]. Among these *MYB* transcription factors, the R2R3-MYB group is the largest transcription factor subfamily in plants [8, 9]. As the R2R3-MYB transcription factor is the largest transcription factor subfamily, genome identification of R2R3-MYB transcription factors has been conducted in several sequenced plants. There are 126 R2R3-MYB transcription factors in *Arabidopsis thaliana* [10], 108 in grape [11], 100 in sweet orange [12], 222 in apple [13] and 192 genes in *Populus trichocarpa* [14].

Most R2R3-MYB proteins among different species are largely conserved, and they can cluster into the same subgroups according to their sequence similarity. However, divergence between them also exists. A comparative analysis of R2R3-MYB genes among different plant species revealed that this transcription factor gene family has undergone extensive expansion during evolution. The expansion of the R2R3-MYB family in plants fits well with the observation that many R2R3-MYB members participate in various biological processes and plant-specific processes [15].

More and more R2R3-MYB transcription factors have been functionally identified since the first identification of the plant *MYB* gene *C1* from *Zea mays* [16]. The R2R3-MYB transcription factors are mainly involved in stamen and pollen maturation [17], trachoma initiation [18], root formation and development [19, 20], hormone signal transduction [21], embryogenesis, and stress [22–24]. Recently, the functions of R2R3-MYB transcription factors in the regulation of secondary metabolism (particularly anthocyanin biosynthesis and metabolism) have been reported in many species, including apple [25], grape [26], litchi [27] and strawberry [28]. Based on phylogenetic analysis, the R2R3-MYB transcription factors associated with anthocyanin biosynthesis in different species typically belong to the same subgroup. In *P. trichocarpa,* the anthocyanin content of male catkins is higher, and the transcript levels of R2R3-MYB transcription factors, including *PtrMYB116*, *PtrMYB117*, *PtrMYB118*, and *PtrMYB119*, in the male catkins of *P. trichocarpa* are also higher. Another R2R3-MYB transcription factor*, PtrMYB120*, could play a role in vegetative tissues by evoking anthocyanin biosynthesis to protect them from the deleterious effects of UV light [14]. Transgenic poplars overexpressing *PtrMYB119*, *PtrMYB120,* or *PdeMYB118* show an elevated accumulation of anthocyanins throughout the plant [29, 30]. Although some R2R3-MYB transcription factors associated with anthocyanin biosynthesis have been identified, many more MYB transcription factors associated with anthocyanin biosynthesis are required to obtain new colored-leaf tree species.

With the development of the social economy, the roles of colored-leaf trees in urban beautification are increasing. Leaf color formation is very complicated, and the distribution and concentration of anthocyanins play important roles in the formation of leaf color. Anthocyanin accumulation is controlled through the coordinated expression of genes encoding the anthocyanin biosynthetic pathway enzymes, and R2R3-MYB transcription factors are considered crucial in the regulation of anthocyanin synthesis.

In the current study, a comprehensive investigation of *PdeMYB* transcription factors in colored-leaf poplar (*Populus deltoids*) was conducted, and the analysis of phylogenetic relationships, sequence features, gene duplication, chromosome distribution, and motif recognition was performed. In addition, a comprehensive expression analysis of *PdeMYB* genes in green leaf poplar (L2025) and colored leaf poplar (QHP) using previous RNA-seq data was performed. The expression levels of candidate *PdeMYB* genes in green leaf poplar (L2025) and colored leaf poplar (CHP) were also evaluated by qRT-PCR analysis. These findings should not only provide a characterization of the *PdeMYB* gene superfamily but also provide valuable information for further functional elucidation of these genes in colored-leaf poplar, which is useful for generating colored-leaf tree species by genetic engineering.

## Results

### Genome-wide identification of the *PdeMYB* gene family in *P. deltoids*

A total of 302 *PdeMYB* genes were identified based on the complete genome sequences of *P. deltoids*. The length of amino acids for the identified *PdeMYB* proteins ranged from 51 bp (*PdeMYB190*) to 1717 bp (*PdeMYB289*), with an average of 342.8 bp (Additional file 1). The molecular weight of the identified *PdeMYB* proteins ranged from 5.6084 kDa (*PdeMYB190*) to 188.4509 kDa (*PdeMYB289*), and the predicted isoelectric points of these ranged from 4.08 (*PdeMYB223*) to 10.98 (*PdeMYB298*). In addition, the subcellular localization of the identified *PdeMYB* proteins was also predicted, and 208 of 302 (approximately 69%) *PdeMYB* proteins were localized in the nucleus (Additional file 1).

In plants, MYB proteins are characterized by a highly conserved MYB domain at the N-terminus, which contains one to four imperfect repeats, and can be classified into four major subfamilies: 1R-like MYB, R2R3-MYB, R1R2R3-MYB, and 4R-MYB. In our study, a total of 302 *PdeMYB* transcription factors were identified, including 183 R2R3-MYB, five R1R2R3-MYB, one 4R-MYB, and 113 1R-MYB.

### Phylogenetic analysis of the *PdeMYB* gene family between *P. deltoids* and *Arabidopsis*

**Fig. 1:**
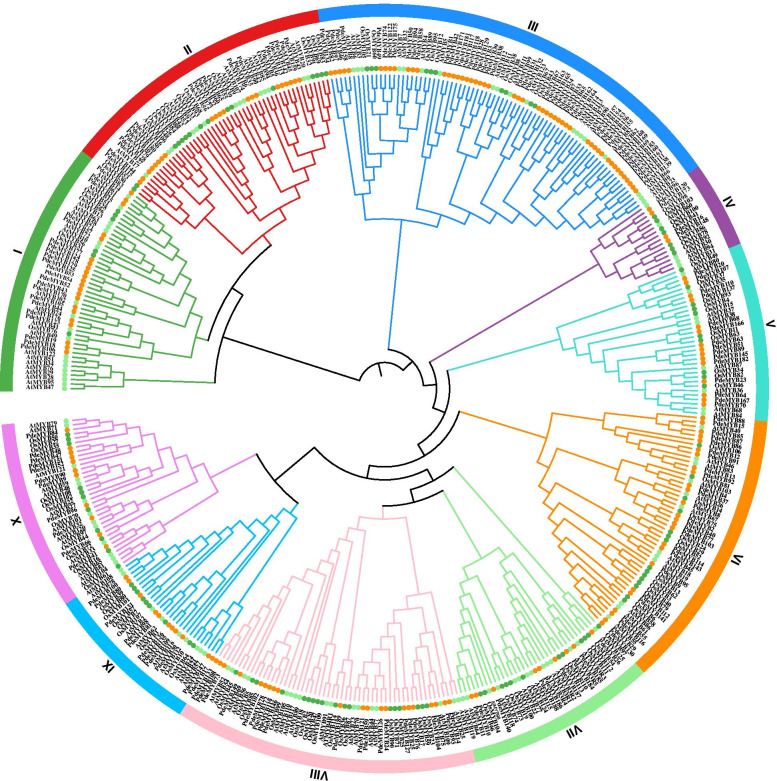
Phylogenetic analysis of R2R3-MYB, R1R2R3-MYB and 4R-MYB gene families in *Arabidopsis*, rice and *P. deltoids*. An un-rooted phylogenetic tree of *MYB* genes in *Arabidopsis*, rice and *P. deltoids* was constructed using the neighbor-joining method in MEGA 7.0 software with a bootstrap test (replicated 1000 times). The *MYB* gene families in *Arabidopsis*, rice and *P. deltoids* were marked light green, dark green and yellow, respectively

An unrooted phylogenetic tree among the R2R3-MYB, R1R2R3-MYB, and 4R-MYB genes of *P. deltoids*, rice, and *Arabidopsis* was constructed using the neighbor-joining method with MEGA 7.0 (Fig. 1). The *PdeMYB* genes of *P. deltoids* were divided into 10 groups (I to X) according to their sequences. Each group contained a different number of *PdeMYB* genes. Group III contained 50 *PdeMYB* genes, which was the largest number in the 10 groups, while group IV contained 4 *PdeMYB* genes, which was the smallest number in the 10 groups. Except group IV, VII, and X, the other groups of *P. deltoids* possessed much more MYB gene members than these of *Arabidopsis* and rice in the seven out of ten groups. Each group could be further divided into several subgroups. Some subgroups just included *P. deltoids MYB* genes but no *AtMYB* and *OsMYB* gene, indicating that these genes may have occurred in *P. deltoids* during evolutionary process. While some subgroups just contained *AtMYB* or *OsMYB* genes with no *P. deltoids MYB* genes, suggesting that some evolutionary changes occurred in the genome – the *MYB* genes could have been either acquired in *Arabidopsis* or rice during evolution or lost in *P. deltoids*. The gain and loss of species-specific *MYB* genes could have resulted in functional divergence.

**Fig. 2:**
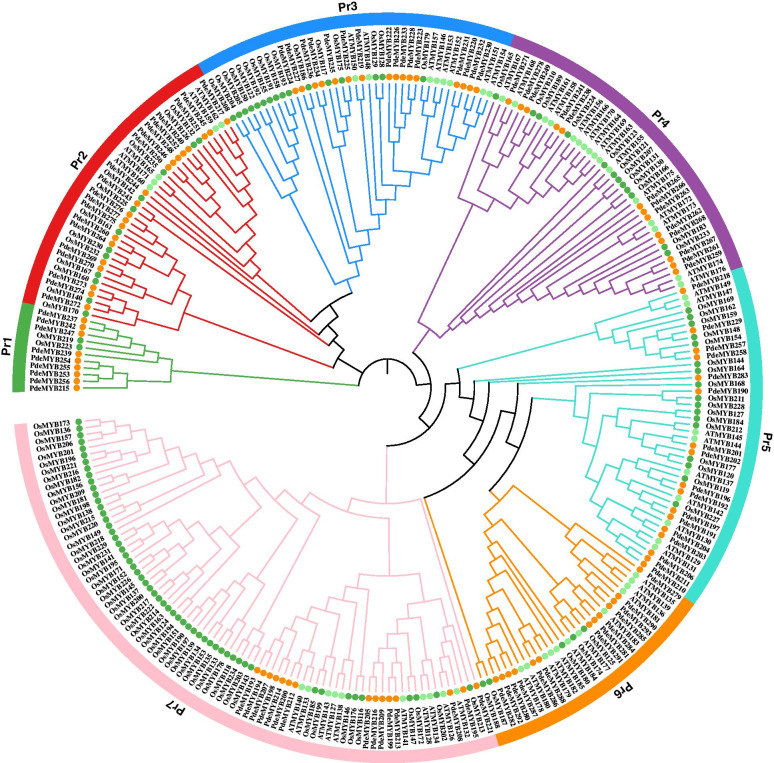
Phylogenetic analysis of 1R-MYB gene families in *Arabidopsis*, rice and *P. deltoids*. An un-rooted phylogenetic tree of *MYB* gene family among *Arabidopsis*, rice and *P. deltoids* was constructed using the neighbor-joining method in MEGA 7.0 software with a bootstrap test (replicated 1000 times). The *MYB* gene families in *Arabidopsis*, rice and *P. deltoids* were marked light green, dark green and yellow, respectively

The phylogenetic tree of 1R-MYB genes in *Arabidopsis*, rice, and *P. deltoids* was constructed using the neighbor-joining method with MEGA 7.0 (Fig. 2). The *PdeMYB* genes in *P. deltoids* were divided into seven groups based on their sequences. Most groups contained the 1R-MYB genes of *P. deltoids*, rice and *Arabidopsis*, while Pr1 group just contained the 1R-MYB genes of *P. deltoids* and *rice*. Pr2 contained 20 *PdeMYB* genes, which was the largest number in the seven groups, while Group Pr1 contained 9 *PdeMYB* genes, which was the smallest number in the seven groups. Except group Pr4 and Pr7, the other groups of *P. deltoids* possessed much more *MYB* gene members than these of *Arabidopsis* and rice in the five out of seven groups. In Pr1 group, one of the subgroups contained all 1R-MYB genes of *P. deltoids* including *PdeMYB215*, *PdeMYB258*, *PdeMYB*253, *PdeMYB255* and *PdeMYB254*. In Pr6 group, one of the subgroups contained all 1R-MYB genes of rice, such as *OsMYB173*, *OsMYB136*, *OsMYB157*, *OsMYB206* and so on. In Pr4 group, one of the subgroups contained all 1R-MYB genes of *Arabidopsis* including *AtMYB156*, *AtMYB163*, *AtMYB164*, *AtMYB166*, *AtMYB169*, and *AtMYB170*. There are much more MYB gene members of rice in Pr7 than these of *P. deltoids* and *Arabidopsis*, which indicated that these *MYB* genes could be either acquired in rice during evolution or lost in *P. deltoids* and *Arabidopsis*. The above results also indicated the differences during the evolutionary process of 1R-MYB genes between *P. deltoids* and *Arabidopsis*.

### Conserved gene structure and protein motif analysis of the *PdeMYB* gene family in *P. deltoids*

**Fig. 3:**
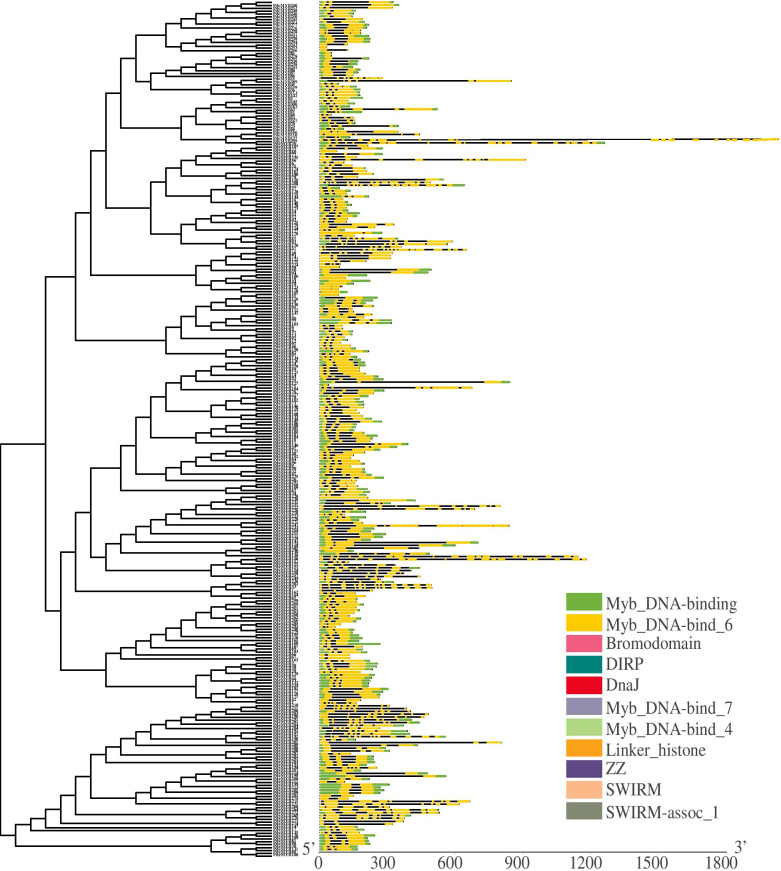
Exon/intron structure of *PdeMYB* genes in *P. deltoids*. The exons are represented by orange round-cornered rectangles. The black lines connecting two exons represent introns

To better understand the structural diversity and motif composition of *PdeMYB* genes, the intron-exon structure pattern was analyzed and visualized using the Gene Structure Display Server 2.0 (Fig. 3). A total of 292 *PdeMYB* genes possessed exons varying from 1 to 10, which accounted for 97% of the total *PdeMYB* genes. Sixteen *PdeMYB* genes lacked introns and had only one exon, including *PdeMYB14*, *PdeMYB24*, *PdeMYB25*, *PdeMYB59*, *PdeMYB76*, *PdeMYB121*, *PdeMYB126*, *PdeMYB127*, *PdeMYB190*, *PdeMYB229*, *PdeMYB248*, *PdeMYB264*, *PdeMYB266*, *PdeMYB267*, *PdeMYB294,* and *PdeMYB295*. The majority (164 of 302) of the *PdeMYBs* had typical splicing, with three exons and two introns. *PdeMYB256* and *PdeMYB257* contained 19 exons and 18 introns, which was the greatest number of exons in the total *PdeMYB* genes. Moreover, the number of introns in the *MYB* genes appeared to be limited, as most *PdeMYB* genes (75%) had no more than two introns. Phylogenetic analysis of the *PdeMYB* gene family was performed according to the intron number and exon length. As the position(s) of the intron(s) were fully conserved, genes in the same subgroups had similar intron patterns, such as in *PdeMYB238*/*PdeMYB246* and *PdeMYB113*/*PdeMYB142* (Fig. 3).

**Fig. 4:**
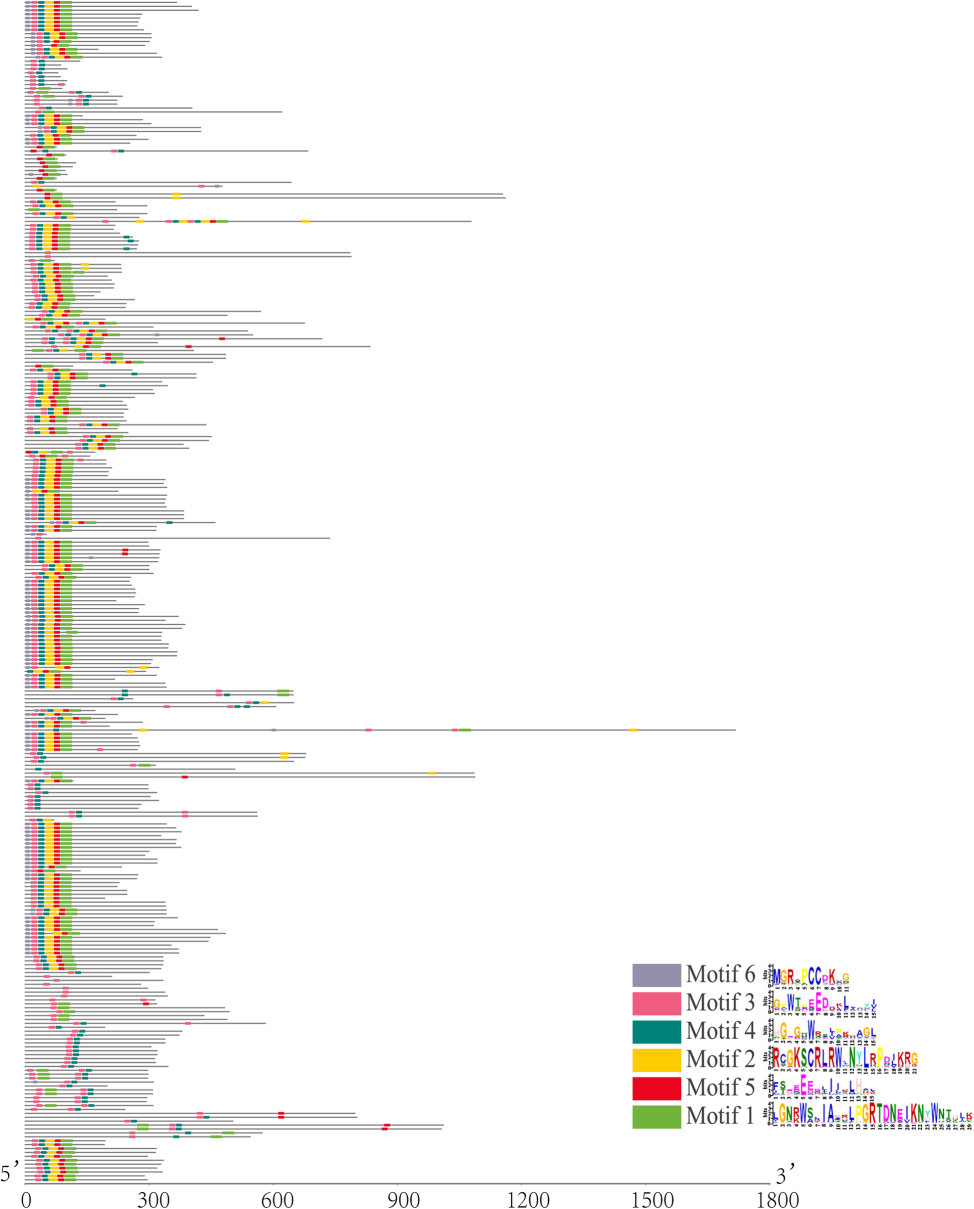
Distribution of conserved motifs in each *PdeMYB* gene. Schematic diagram of motif structure in *P. deltoids PdeMYB* gene family using MEME. The relative positions of each conserved motif within the *PdeMYB* proteins are shown in color. The black lines represent the non-conserved sequences. The scale bar represents 200 aa

As shown in Fig. 4, six conserved motifs were predicted to further reveal the diversification of *PdeMYB* genes in *P. deltoids*. In most cases, a motif is repeated only once or twice, and one gene has only one motif repeated. However, only a few special cases exist. *PdeMYB71*, *PdeMYB194*, *PdeMYB170,* and *PdeMYB83* contained two repeated motifs, and each motif was repeated twice. *PdeMYB6* contained three repeated motifs: motif 3 repeated three times, motif 4 repeated twice, and motif 6 repeated four times. *PdeMYB57* also contained three repeated motifs, and each motif was repeated twice. *PdeMYB21* contained four repeated motifs: motif 2 repeated twice, motif 3 repeated twice, motif 4 repeated twice, and motif 5 repeated twice.

### Chromosomal location and duplication events of *PdeMYBs* in *P. deltoids*

**Fig. 5:**
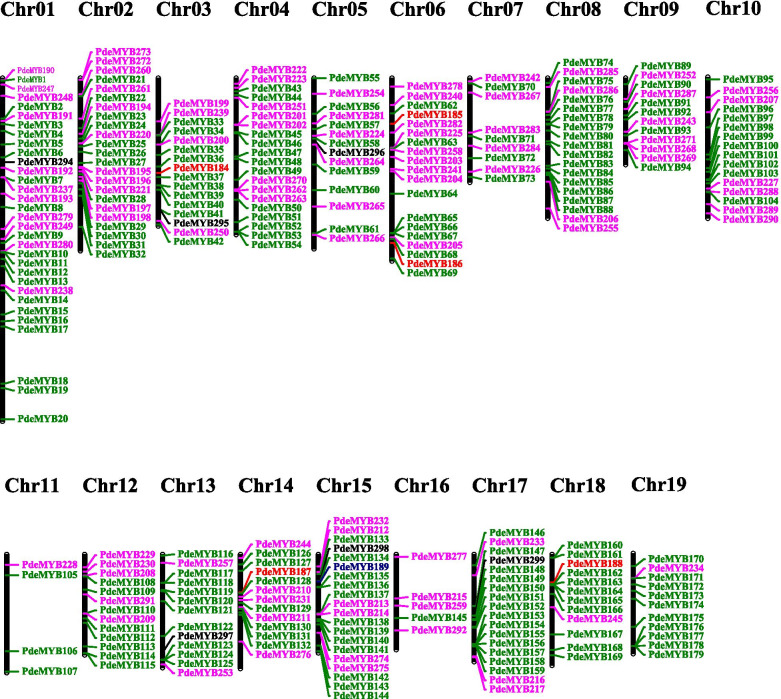
Chromosomal locations of *PdeMYB* genes in *P. deltoids*. There are 289 *PdeMYB* genes mapped on 19 chromosomes, and the other 13 *PdeMYB* genes belonged to unassembled scaffolds. The chromosomal position of each *PdeMYB* gene was mapped according to the *P. deltoids* genome. The chromosome number is indicated at the top of each chromosome

To determine the genomic distribution of the *PdeMYB* gene family in *P. deltoids*, the chromosomal locations of *PdeMYBs* were evaluated according to their genomic sequences. There are 19 chromosomes in the *P. deltoids* genome, and each of the 19 *P. deltoids* chromosomes contained *PdeMYB* genes (Fig. 5). Although *PdeMYB* genes were distributed on all chromosomes in *P. deltoids*, their distribution on each chromosome seemed to be uneven. In the present results, there were 289 *PdeMYB* genes assigned to 19 chromosomes: 32 *PdeMYBs* were present on chromosome 1; 23 on chromosome 2; 20 on chromosomes 4 and 15; 19 on chromosomes 6 and 8; 18 on chromosome 17; 16 on chromosomes 3 and 10; 14 on chromosome 5; 13 on chromosomes 12, 13, and 14; 12 on chromosomes 9 and 18; 11 on chromosome 19; 9 on chromosome 9; 5 on chromosome 16; and 4 on chromosome 11. Chromosome 1 had the highest number of *PdeMYB* genes (32), followed by chromosome 2 (23). Chromosome 11 had the least number of *PdeMYB* genes (4). In addition, 13 *PdeMYB* genes belonged to the scaffold.

Gene duplication, especially tandem and segmental duplication events, contributes greatly to the diversity and evolution of gene families. In the present study, there were 59 duplicated *PdeMYB* gene pairs in the *P. deltoids* genome, and most of them belonged to the segmental duplication or whole genome duplication event. A total of 56 pairs of *PdeMYB* genes were identified as whole genome duplication or segmental duplications, including 53 duplication events between different chromosomes as well as three duplication events within the same chromosome (*PdeMYB10*/*PdeMYB11*, *PdeMYB12*/*PdeMYB13*, and *PdeMYB47*/*PdeMYB48*), while three pairs of *PdeMYBs* were found as tandem repeats in *P. deltoids*, including *PdeMYB156*/*PdeMYB157*, *PdeMYB53*/*PdeMYB54*, and *PdeMYB155*/ *PdeMYB156*. Interestingly, genes such as *PdeMYB156*, *PdeMYB277*, *PdeMYB162*, and *PdeMYB47* occurred in more than two gene pairs (Additional file 2).

To evaluate the selection of the duplicated *PdeMYB* gene pairs, the non-synonymous to synonymous substitution ratios (Ka/Ks) were calculated according to the whole genome analysis of gene duplications. When the Ka/Ks ratio is greater than one, the identified genes are under positive selection; when the Ka/Ks ratio is one, the identified genes are under neutral selection; when the Ka/Ks ratio is less than one, the identified genes are under negative purifying selection. In the present study, there were 57 *PdeMYB* gene duplicated pairs, the Ka/Ks ratios of which were less than one, indicating that these genes are under negative purifying selection and contribute largely to the maintenance of function in the *PdeMYB* gene family of *P. deltoids*. There were two *PdeMYB* gene duplicated pairs, the Ka/Ks ratios of which were more than one, indicating that these genes have likely experienced positive selection. Ks was used as a proxy for time to estimate the dates of the duplication event. The segmental duplicated events in *P. deltoids* appeared to have occurred from 0.001264253 (Ks= 3.79E-05) to 50.944 mya (Ks= 1.52832). The Ks of tandem duplication of *PdeMYB* genes occurred from 1.823416667 to 7.34143333 mya (Additional file 2).

**Fig. 6:**
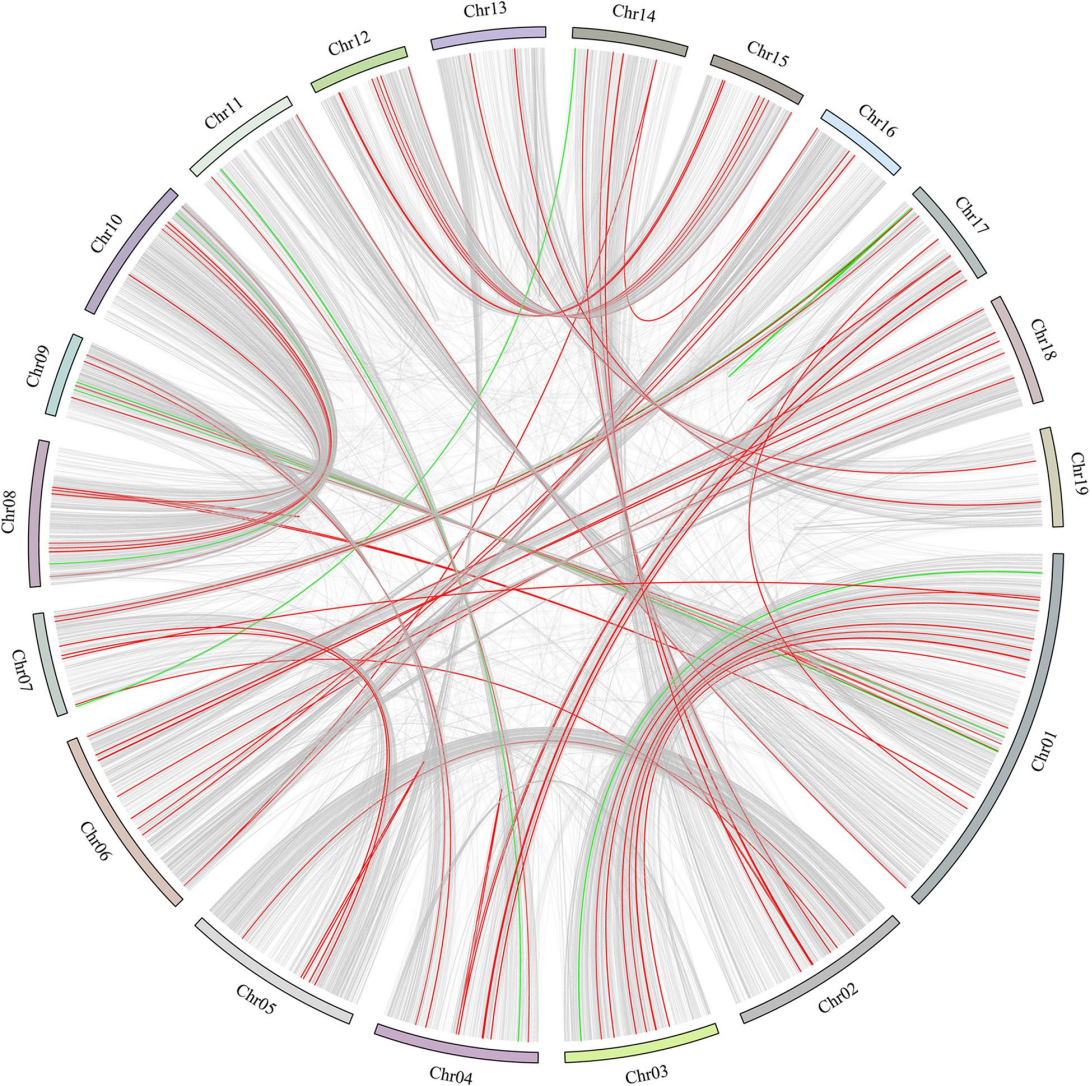
Chromosomal localization and paralogs of *PdeMYB* genes in *P. deltoids*. The chromosomal position of each *PdeMYB* was mapped according to the *P. deltoids* genome. A red link indicates that two genes belong to the *PdeMYB* gene family, a green link indicates that one of the genes belongs to the *PdeMYB* gene family, and a gray link indicates that none of the genes belong to the *PdeMYB* gene family

To evaluate the possible relationship between the *PdeMYB* genes and potential duplication events, the collinearity of the *PdeMYB* gene family in *P. deltoids* was identified using the BLASTP and MCScanX methods. A total of 56 segmental duplication events with 102 *PdeMYB* genes were identified in the *P. deltoids* genome (Fig. 6). *PdeMYB* genes were located within synteny blocks on all chromosomes. Intrachromosomal duplication was also observed in the *P. deltoids* genome.

**Fig. 7:**
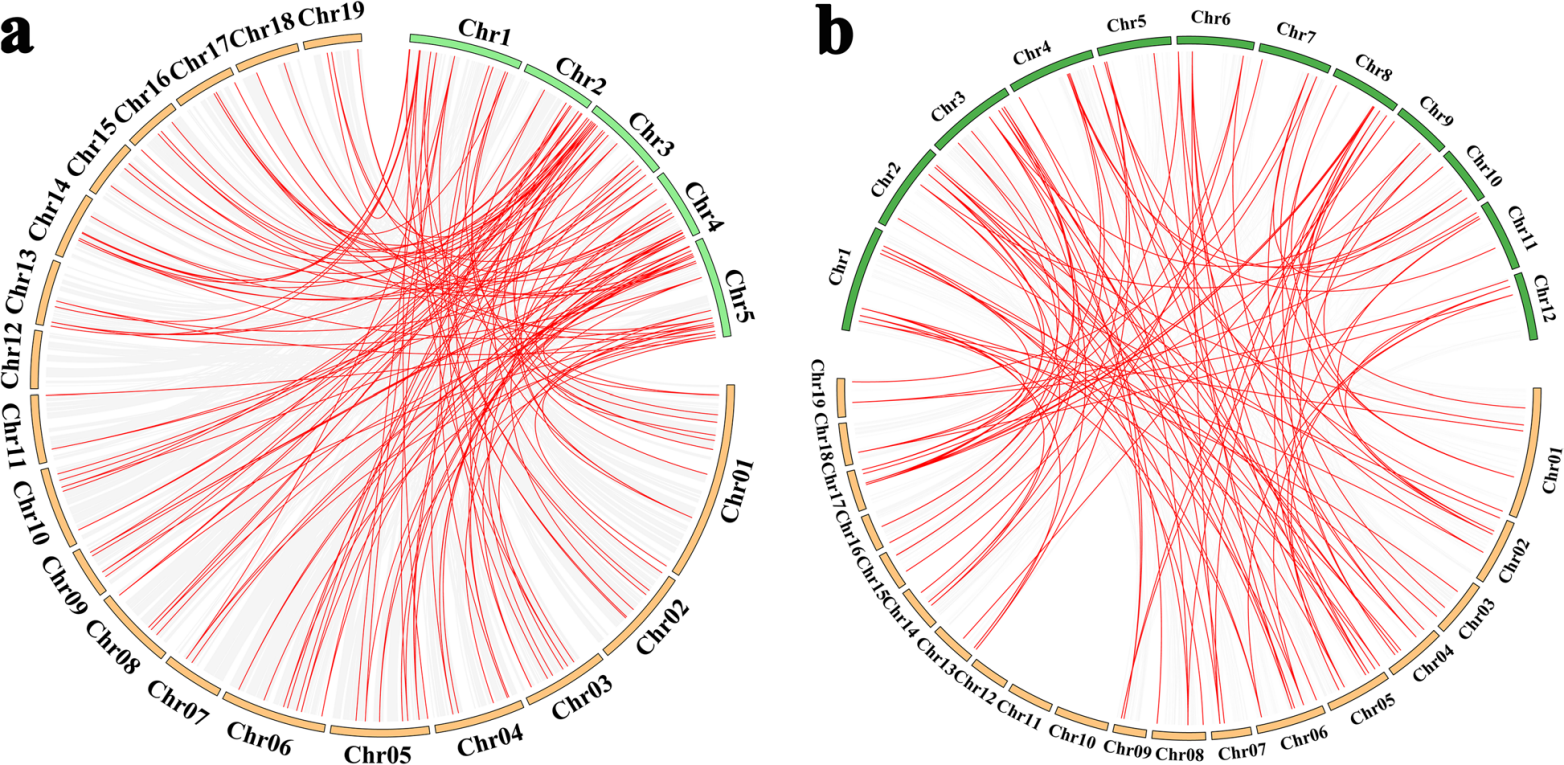
Gene duplication and synteny analysis of *MYB* genes between *P. deltoids, Arabidopsis*, and *rice*. a Gray link in the background indicates the collinear blocks within *P. deltoids* and *Arabidopsis* genomes, while the red link highlights the syntenic *MYB* gene pairs. b Gray link in the background indicates the collinear blocks within *P. deltoids* and rice genomes, while the red lines highlight the syntenic *MYB* gene pairs

To further evaluate the potential evolutionary mechanisms of *the PdeMYB* gene family in *P. deltoids*, two comparative syntenic maps were constructed between *P. deltoids*, *Arabidopsis*, and rice (Fig. 7). There were 60 collinear *MYB* gene pairs between *P. deltoids* and *Arabidopsis* and 77 orthologs between *P. deltoids* and rice. The details of the collinear *MYB* gene pairs were provided in Additional files 3 and 4. The number of orthologous events of *PdeMYB*-*OsMYB* was much greater than that of *PdeMYB*-*AtMYB* as there were much bigger genome size and much more chromosome numbers.

### Expression profile of the *PdeMYB* genes in QHP and L2025 by RNA-seq

**Fig. 8:**
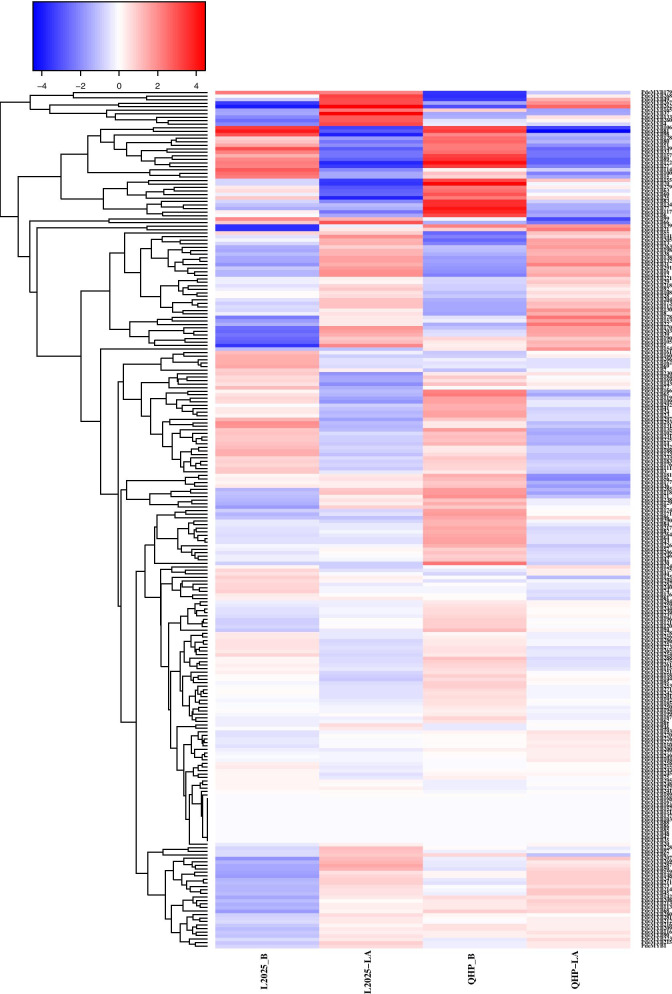
Expression profile of *PdeMYB* genes in the leaves and buds of the QHP and L2025. QHP-L, QHP leaf; L2025-L, L2025 leaf; QHP-B, QHP bud; L2025-B, L2025 bud

To evaluate the expression pattern of *PdeMYB* genes in colored-leaf poplar, the expression profiles of *PdeMYB* genes in the leaves and buds of QHP and L2025 were screened from the previous data. The expression levels of candidate *PdeMYB* genes in the leaves of QHP and L2025 were also evaluated (Fig. 8). The expression level of candidate *PdeMYB* genes in the leaves of QHP was more than 10 times that in L2025 or specifically expressed in the leaves of QHP or L2025, and these candidate *PdeMYB* genes are shown in Additional file 5. The expression level of *PdeMYB154* in the leaves of QHP was 13.28 times higher than that of L2025, and 33.21 times higher for *PdeMYB155*. Many *PdeMYB* transcription factors are specifically expressed in QHP, such as *PdeMYB25*, *PdeMYB27*, *PdeMYB60*, *PdeMYB114*, and *PdeMYB160*, which indicated that these genes might play important roles in the formation of colored leaves in QHP.

### Expression analyses of *PdeMYB* genes in CHP and L2025 by qRT-PCR analysis

**Fig. 9:**
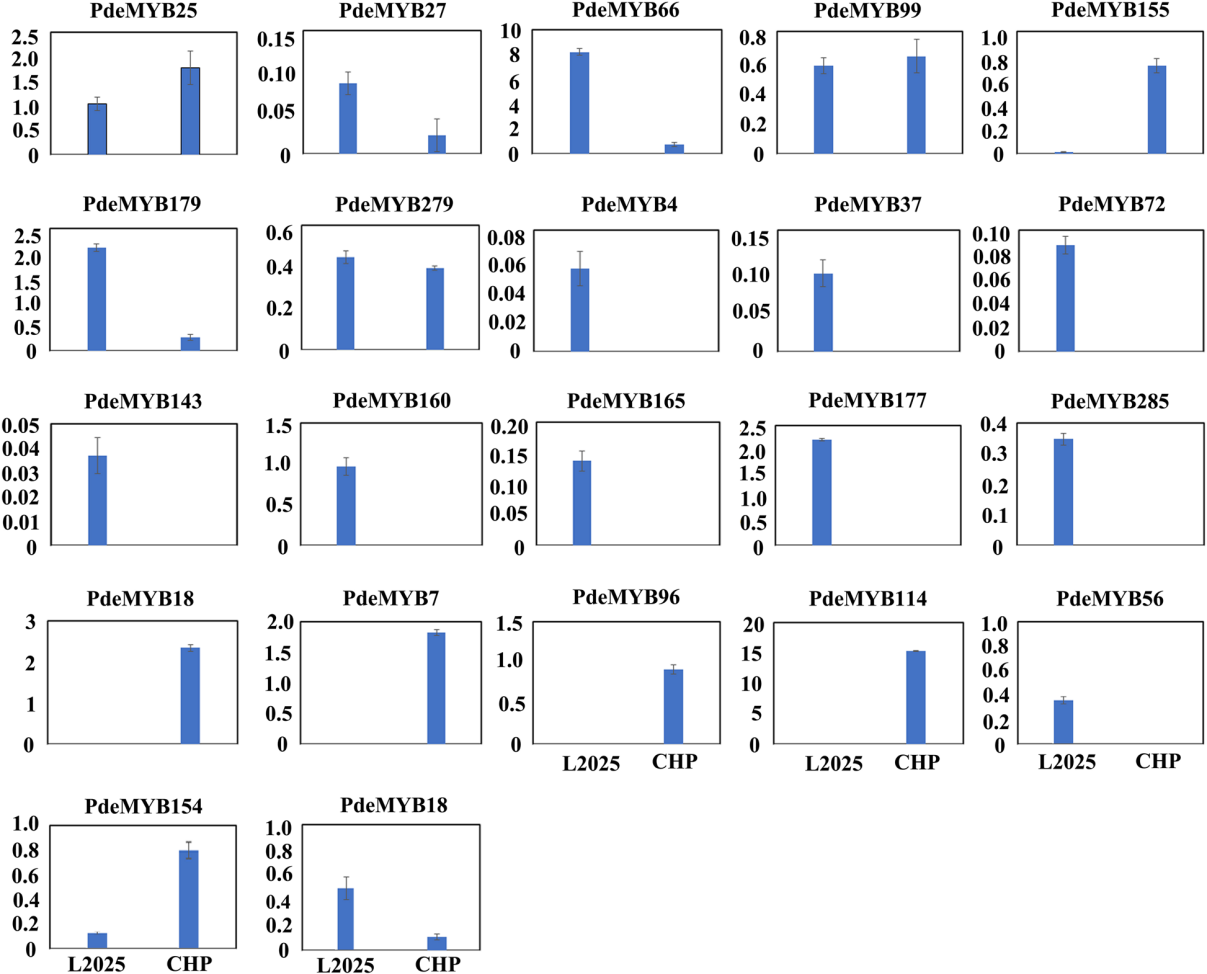
The relative expression level of candidate *PdeMYB* genes in the leaves of L2025 and CHP. Bars indicate the mean ± SE, n = 3

To better explore the expression pattern of *PdeMYB* genes in leaf-colored poplar, the candidate *PdeMYB* genes were also evaluated in the leaves of CHP and L2025 (Fig. 9). The expression levels of some *PdeMYB* genes between CHP and L2025 were not substantially different, such as *PdeMYB25*, *PdeMYB99*, and *P9deMYB279*, which may not be important in the coloration of CHP. Some *PdeMYB* genes such as *PdeMYB60*, *PdeMYB70*, *PdeMYB96,* and *PdeMYB114*, which are specifically expressed in the leaves of CHP, may be candidate *PdeMYB* genes to further explore the mechanism of leaf coloration in poplar. Other *PdeMYB* genes, such as *PdeMYB4*, *PdeMYB37*, *PdeMYB72,* and *PdeMYB146*, which are specifically expressed in the leaves of L2025, may also be important candidate genes for studying their functions.

## Discussion

*MYB* transcription factors play important roles in secondary metabolism (especially in the anthocyanin pathway), development, signal transduction, and disease resistance, and comprise one of the largest transcription factor families in plants [7]. Many reports have indicated that MYB transcription factor genes can regulate the formation of leaf coloration in plants. Transgenic *Arabidopsis* overexpressing *PAP1*/*AtMYB75* increases the expression level of structural genes associated with anthocyanin biosynthesis, which contributes to higher anthocyanin accumulation [7, 31, 32]. Tobacco overexpressing *IbMYB1a* can upregulate the expression levels of several structural genes, such as *ANS* and *DFR* 22, which can promote the accumulation of anthocyanin [33]. Tobacco overexpressing *CsMYB6A* increases the expression levels of structural genes associated with flavonoid synthesis, such as *3GT* and *CHS*, leading to a high accumulation of anthocyanins in the leaves of transgenic tobacco [34]. Transgenic poplar overexpressing *MYB6* shows a red color in the young leaves and shoots by increasing their anthocyanin accumulation [35]. *PtrRML1*, a repressor motif-containing poplar R3 MYB-like transcription factor, the overexpression of which in transgenic Arabidopsis can reduce their anthocyanin content in stems, petioles, and rosette leaves [36]. Overexpression of *PtoMYB156*, *PtrMYB57*, *MYB182*, *MYB165*, and *MYB194* separately can also downregulate anthocyanin biosynthesis in transgenic poplar [37–40]. Transgenic poplar overexpressing *PtrMYB119* and *PdeMYB118* separately has higher anthocyanin content in leaves compared with that in wild-type poplar [29, 30]. Although some MYB genes from poplar can regulate the biosynthesis of anthocyanin, many more *MYB* genes are needed to regulate the leaf color of poplar. To date, only *PtrMYB119* and *PdeMYB118* from poplar have been shown to change the leaf color of poplar under normal conditions. Therefore, genome-wide analysis of the *MYB* family genes in poplar was characterized, and many more *MYB* family genes from poplar associated with anthocyanin biosynthesis need to be identified in the future.

Many *MYB* gene families have been identified in different plant species. There are 198, 233, and 197 *MYB* genes in *Arabidopsis*, rice, and soybean, respectively [41, 42]. In the present study, 302 *PdeMYB* transcription factors were identified in the *P. deltoids* genome. Among these, R2R3-MYBs accounted for 61% of the identified MYB transcription factors, and MYB-related proteins were the second largest subfamily of MYB proteins, accounting for 37% in our study, which is similar to the fraction of MYB-related proteins in rice (40%) [43]. In *P. trichocarpa*, there are 192 R2R3-MYB and 5 R1R2R3-MYB [14], which is similar to our results in the *P. deltoids* genome.

Gene duplication plays an important role in gene family expansion in plants [44], either in the form of segmental duplication, tandem duplication, or transposition events [45]. When gene duplication occurs within the same chromosome, tandem duplication occurs. In different chromosomes, this is considered segmental duplication. Both segmental and tandem duplication can lead to the diversification of species, which might also be crucial for increasing the adaptability of plants to different environmental conditions [46]. In the present study, expansion of the R2R3-MYB gene family in *P. deltoids* resulted from both segmental duplication and tandem duplication events, which are similar to those in maize and *Gossypium raimondii* [45, 47]. There were three tandem duplications and 56 segmental duplications for *PdeMYB* genes in *P. deltoids*, which indicated that segmental duplication events were a major cause of the expansion of *PdeMYB* genes.

It is generally believed that *PdeMYB* members that fall within certain clades may have common evolutionary origins and conserved functions. Therefore, the putative functions of the *P. deltoids* MYB proteins can be speculated through the functional clades of *Arabidopsis* MYBs. Phylogenetic analyses and evolutionary relationships of the *PdeMYB* gene family have been systematically studied among different species, suggesting the conservation and expansion of *P. deltoids MYBs*. In addition, the pattern of gene structures can be used to evaluate the phylogenetic relationships in a gene family. The number of exons in the 302 *PdeMYB* genes ranged from one to ten, and most of the *PdeMYBs* had three exons and two introns, which is similar to the results in other plants [9]. Remarkably, the *PdeMYB* members within the same subfamily shared similar exon/intron patterns [11]. In our study, most of the *PdeMYB* genes were disrupted by no more than two introns, which is consistent with previous reports that most MYB-related genes in land plants contain up to two introns [48].

Phylogenetic classification of genes into subfamilies was performed according to the gene structure and domain analysis. In general, members of the same subfamily often share similar sequences, conserved motifs, and even binding partners, and are likely to share similar functions [49, 50]. Therefore, when there is a lack of sufficient data, functional characterization of unknown proteins is generally carried out based on comparisons with annotated proteins [13, 51]. In the present study, *PdeMYB* genes were clustered into the same group as those in *Arabidopsis thaliana*, indicating the functional conservation of R2R3-MYBs between species. In *Arabidopsis thaliana*, *MYB75*, *MYB90*, *MYB113*, and *MYB114* have been reported to be involved in the regulation of anthocyanin biosynthesis [7, 10, 51]. *PdeMYB155*, *PdeMYB157*, *PdeMYB156*, *PdeMYB153,* and *PdeMYB154* were clustered into the same group as *AtMYB75*, *AtMYB90*, *AtMYB113,* and *AtMYB114* in *Arabidopsis thaliana*, indicating that these genes in poplar might be involved in the synthesis of anthocyanin. In accordance with this, the expression level of *PdeMYB154* and *PdeMYB155* in the leaves of QHP was much higher than these in L2025 (Fig. 8), and a similar situation occurred in another colored leaf cultivar, CHP (Fig. 9). *PdeMYB60* and *PdeMYB114* are specially expressed in the leaves of QHP and CHP plants, indicating that they may play important roles in leaf coloration in poplar (Figs. 8 and 9). In contrast to the expression patterns of *PdeMYB60* and *PdeMYB114* in QHP and CHP, *PdeMYB25* was specifically expressed in the leaves of QHP, but there was no significant difference in its expression level between CHP and L2025 (Figs. 8 and 9), which indicated that *PdeMYB25* might not be a critical gene during leaf coloration in poplar. Although many genes might be involved in leaf coloration in poplar, the detailed functions of these anthocyanin-related genes should be further identified by experimental assays in the future.

## Methods

### Identification of the *PdeMYB* gene family in *P. deltoids*

The hidden Markov Model profile of MYB binding domain with accession number was obtained from the Pfam database (http://pfam.xfam.org/) [52], which was used to search candidate *PdeMYB* genes from *P. deltoids* genome with HMMER 3.1, with a cutoff value of 0.01. The *P. deltoids* genome was downloaded from the Joint Genome Institute’s Plant Genomics Portal (https://phytozome-next.jgi.doe.gov/), and HMMER 3.1 was downloaded from http://hmmer.org/download.html. In addition, *MYB* genes in *Arabidopsis thaliana* and rice were used to further identify candidate *PdeMYB* genes in *P. deltoids* genome, which were downloaded from the *Arabidopsis* Information Resource (TAIR; http://www.arabidopsis.org/) and RGAP release 7 (http://rice.plantbiology.msu.edu/). The SMART and NCBI-CDD were used to confirm the acquired *Pde*MYB proteins in *P. deltoids*, the website of which were http://smart.embl-heidelberg.de and http://www.ncbi.nlm.nih.gov/Structure/cdd/wrpsb.cgi.

### Sequence analysis and structural characterization of *PdeMYB* genes in *P. deltoids*

Based on the conserved domains and genome sequence of the *PdeMYB* genes, the exon-intron organization of the *PdeMYB* genes, including intron distribution patterns, phases, and intron-exon boundaries, was graphically displayed by the Gene Structure Display Server GSDS2.0 (http://gsds.cbi.pku.edu.cn/). The conserved motifs of the *PdeMYB* transcription factors were predicted using a MEME Suite analysis [53], and the software was downloaded from http://meme-suite.org/tools/meme. The maximum number of motifs was set to identify 20 motifs, and the optimum width of motifs was set from 6 to 100 amino acids.

### Chromosome distribution, gene duplication, and synteny of *PdeMYB* genes in *P. deltoids*

The chromosome distribution of *PdeMYB* genes was obtained from the database of the *P. deltoids* genome (https://phytozome-next.jgi.doe.gov/), and the chromosomal locations of the *PdeMYB* genes were visualized using MapChart software [54]. Segmental duplication, tandem duplication, and synteny blocks of the orthologous *PdeMYB* genes between *P. deltoids* and *Arabidopsis* as well as *P. deltoids* and rice were evaluated using MCscanX (Multiple Collinearity Scan toolkit; http://chibba.pgml.uga.edu/mcscan2/) [55]. The synonymous (Ks) and nonsynonymous (Ka) substitutions of *PdeMYB* genes were calculated using KaKs_Calculator 2.0 to estimate their duplication events [56], and circos v0.69 was used to graphically present the synteny blocks of the orthologous *PdeMYB* genes between *P. deltoids* and *Arabidopsis*, *P. deltoids*, and rice [57].

### Phylogenetic analysis and classification of *PdeMYB* proteins in *P. deltoids*

Phylogenetic analysis was performed using the full-length amino acid sequences of *PdeMYB*, *OsMYB*, and *AtMYB* proteins. An unrooted neighbor-joining phylogenetic tree was constructed through multiple sequence alignments of these *MYB* proteins using MEGA 7.0, and these *MYB* proteins were classified into different groups based on the topology of the phylogenetic tree. The parameters were as follows: pairwise deletion, Poisson model, and 1000 bootstrap replications [58]. The data matrices and resulting trees were deposited in TreeBase (http://purl.org/phylo/treebase/ phylows/study/TB2:S28602 and http://purl.org/phylo/treebase/ phylows/ study/TB2:S28603), and the accession numbers are 28602 and 28603, respectively.

### Expression analyses of *PdeMYB* genes in Quanhong poplar (QHP) and *Populus* sp. Linn. 2025 (L2025) by RNA-seq

To explore the expression pattern of *PdeMYB* genes in colored-leaf polar QHP and green leaf poplar L2025, the expression profiles of the putative *PdeMYB* genes in the leaves and buds of the QHP and L2025 were retrieved from previous research with *PdeMYB* gene IDs as the queries [59]. The transcript abundance of *PdeMYB* genes was calculated as fragments per kilobase of exon model per million mapped reads (FPKM). The log_2_^(FPKM +1)^ from the RNA-seq data were subjected to hierarchical clustering with Cluster 3.0, and the results were graphically displayed using Java TreeView [53].

### Expression analyses of *PdeMYB* genes between CHP and L2025 by qRT-PCR analysis

Colored leaf poplar with bright red leaves, CHP, and green leaf poplar, L2025, were cultivated in the experimental field of the Nanjing Botanical Garden Mem. Sun Yat-Sen (32°3′N, 118°49′E). To better identify the candidate *PdeMYB* genes associated with leaf color, the expression level of candidate *PdeMYB* genes in previous research that showed significant changes between QHP and L2025 were further evaluated. The leaves of CHP and L2025 were collected in August 2020, and the RNA was extracted using an RNA Aprep Pure Plant Kit (Tiangen, Beijing, China). The quality and concentration of each RNA sample was evaluated by gel electrophoresis and using a NanoDrop 2000 spectrophotometer (Thermo Fisher Scientific, Waltham, MA, USA), and the higher quality RNA with a 260/280 ratio of 1.8-2.1, 260/230 ratio≥2.0, were stored at −80 °C for further analyses. cDNA was synthesized using a ReverTra Ace qPCR RT kit (TOYOBO, Osaka, Japan). The expression levels of candidate *PdeMYB* genes in the leaves of CHP and L2025 were evaluated by qRT-PCR using an Applied Biosystems 7500 Real-Time PCR system (Applied Biosystems, Waltham, MA, USA). Gene-specific primers were designed according to the sequence of candidate *PdeMYB* genes (Additional file 6), and the *ACTIN2* gene was used as a control gene [29]. The thermal cycling conditions were as follows: 95 °C for 2 min, followed by 40 cycles of 95 °C for 5 s, and products collected at 60 °C for 34 s. The relative expression levels of genes were evaluated by the 2^−ΔΔCt^ method [60], and analyzed using SPSS 17.0, with three biological replicates.

## Conclusions

In the present study, 302 *PdeMYB* transcription factors identified in *P. deltoids*. Genomic localization and paralogs of *PdeMYB* genes mapped 289 genes on 19 chromosomes, with collinearity relationships among genes. The conserved domain, gene structure, and evolutionary relationships of *PdeMYBs* were also established and analyzed. The expression levels of some *PdeMYB* genes were significantly different among different colored-leaf poplar varieties, which provided valuable clues for further functional characterization, in addition to providing candidate genes for the future improvement of leaf colorization in *P. deltoids*.

## Supplementary Information

**Additional file 1. **Information of *PdeMYB* genes identified in *Populus deltoids.*

**Additional file 2. **Segmentally and tandemly duplicated *PdeMYB* gene pairs.

**Additional file 3. **One-to-one orthologous relationships between *Populus deltoids* and *Arabidopsis*.

**Additional file 4. **One-to-one orthologous relationships between *Populus deltoids* and *Oryza sativa*.

**Additional file 5.**The candidate *PdeMYB* genes with their expression level in the leaves of QHP more than ten times than these in L2025 or specifically expressed in the leaves of QHP or L2025.

**Additional file 6.** Specific primers used in relative quantitative real-time RT-PCR.

## Data Availability

All data generated or analysed during this study are included in this published article and its supplementary information files. The phylogenetic matrix and trees are available in the TreeBASE repository for Figs. 1 and 2. The links for the phylogenetic matrix and trees of Figs. 1 and 2 are as follows: http://purl.org/phylo/treebase/phylows/study/ TB2:S28602 and http://purl.org/phylo/treebase/phylows/study/TB2: S28603, and the accession numbers are 28602 and 28603, respectively.
