# Supplementary material for: Genome-wide analysis and expression profiles of PdeMYB transcription factors in colored-leaf poplar (Populus deltoids)
Source: BMC Plant Biol. 2021 Sep 23;21:432. doi: 10.1186/s12870-021-03212-1 (PMC8459500; doi:10.1186/s12870-021-03212-1)
Supplement: Supplementary file 1 — Additional file 1. Information of PdeMYB genes identified in Populus deltoids. [file 12870_2021_3212_MOESM1_ESM.docx]

**Additional file 1**. Information of *PdeMYB* genes identified in *Populus deltoids.*

| **Gene name** | **Gene ID** | **Chr.** | **Start site (bp)** | **End site (bp)** | **strand** | **Exon** | **Protein Length (AA)** | **Mw(kDa)** | **pI** | **Predicted**  **subcellular**  **localization** |
| --- | --- | --- | --- | --- | --- | --- | --- | --- | --- | --- |
| PdeMYB1 | Podel.01G005800 | Chr01 | 391697 | 393733 | + | 2 | 317 | 35445.5 | 8.22 | SP |
| PdeMYB2 | Podel.01G078600 | Chr01 | 6263943 | 6265120 | - | 2 | 341 | 38527.3 | 7.12 | Nuclear |
| PdeMYB3 | Podel.01G090000 | Chr01 | 7121616 | 7122966 | + | 2 | 277 | 30818.5 | 9.06 | Nuclear |
| PdeMYB4 | Podel.01G103800 | Chr01 | 8180000 | 8182289 | + | 3 | 310 | 34914.8 | 5.36 | SP |
| PdeMYB5 | Podel.01G126300 | Chr01 | 10252713 | 10254758 | - | 2 | 271 | 30642.1 | 5.09 | Nuclear |
| PdeMYB6 | Podel.01G150900 | Chr01 | 12261956 | 12263749 | + | 2 | 337 | 37688.7 | 6.64 | Nuclear |
| PdeMYB7 | Podel.01G180400 | Chr01 | 15293669 | 15294961 | + | 3 | 195 | 22475.4 | 9.2 | Nuclear |
| PdeMYB8 | Podel.01G209300 | Chr01 | 19949503 | 19951526 | - | 3 | 368 | 40789.3 | 6.77 | SP |
| PdeMYB9 | Podel.01G246300 | Chr01 | 25498767 | 25500345 | - | 3 | 242 | 27350.7 | 8.2 | Nuclear |
| PdeMYB10 | Podel.01G261600 | Chr01 | 26875094 | 26877972 | - | 3 | 484 | 53301.3 | 6.67 | Nuclear |
| PdeMYB11 | Podel.01G262600 | Chr01 | 26951249 | 26954127 | - | 3 | 484 | 53414.5 | 6.84 | Nuclear |
| PdeMYB12 | Podel.01G274500 | Chr01 | 27967180 | 27969731 | - | 2 | 334 | 37178.3 | 5.61 | Nuclear |
| PdeMYB13 | Podel.01G283000 | Chr01 | 29079140 | 29081549 | + | 2 | 328 | 37029.2 | 6.35 | Nuclear |
| PdeMYB14 | Podel.01G319400 | Chr01 | 32827897 | 32828986 | - | 1 | 245 | 26553.1 | 9.7 | Nuclear |
| PdeMYB15 | Podel.01G356800 | Chr01 | 36556252 | 36557828 | - | 3 | 283 | 32668.3 | 5.28 | Nuclear |
| PdeMYB16 | Podel.01G362900 | Chr01 | 37588583 | 37590360 | + | 3 | 320 | 35730.6 | 6.26 | Nuclear |
| PdeMYB17 | Podel.01G369200 | Chr01 | 38395923 | 38398895 | - | 3 | 191 | 21630.1 | 7.03 | SP |
| PdeMYB18 | Podel.01G439000 | Chr01 | 47242441 | 47243880 | + | 4 | 341 | 38462.9 | 7.92 | Nuclear |
| PdeMYB19 | Podel.01G444400 | Chr01 | 47953030 | 47954518 | + | 4 | 328 | 36964.3 | 9.6 | Nuclear |
| PdeMYB20 | Podel.01G492000 | Chr01 | 52721908 | 52723496 | + | 3 | 328 | 36434 | 7.05 | Nuclear |
| PdeMYB21 | Podel.02G040700 | Chr02 | 2803639 | 2805669 | - | 3 | 273 | 30935.6 | 6.24 | Nuclear |
| PdeMYB22 | Podel.02G105600 | Chr02 | 7737396 | 7738835 | + | 3 | 334 | 37083.6 | 6.92 | Nuclear |
| PdeMYB23 | Podel.02G124300 | Chr02 | 9390206 | 9392002 | + | 3 | 346 | 38626.2 | 5.79 | Nuclear |
| PdeMYB24 | Podel.02G134300 | Chr02 | 10069799 | 10071838 | - | 1 | 308 | 33348.1 | 9.28 | SP |
| PdeMYB25 | Podel.02G141800 | Chr02 | 10598223 | 10600555 | + | 1 | 238 | 26292.1 | 8.22 | SP |
| PdeMYB26 | Podel.02G154600 | Chr02 | 11391556 | 11392941 | + | 3 | 309 | 34755 | 6 | Nuclear |
| PdeMYB27 | Podel.02G174500 | Chr02 | 13115392 | 13123188 | + | 3 | 316 | 35014.5 | 7.7 | Nuclear |
| PdeMYB28 | Podel.02G193100 | Chr02 | 14860284 | 14862088 | - | 2 | 286 | 32148.9 | 9.1 | Nuclear |
| PdeMYB29 | Podel.02G205100 | Chr02 | 16279579 | 16281716 | - | 3 | 484 | 53903.6 | 7.71 | Nuclear |
| PdeMYB30 | Podel.02G213100 | Chr02 | 17221604 | 17223121 | + | 3 | 295 | 33449.1 | 5.17 | Nuclear |
| PdeMYB31 | Podel.02G220800 | Chr02 | 18201052 | 18204271 | + | 3 | 402 | 44356.1 | 4.9 | Nuclear |
| PdeMYB32 | Podel.02G251800 | Chr02 | 23032799 | 23035035 | + | 3 | 488 | 54053.6 | 6.99 | SP |
| PdeMYB33 | Podel.03G067500 | Chr03 | 8877447 | 8878770 | - | 3 | 195 | 22519.5 | 10.12 | Nuclear |
| PdeMYB34 | Podel.03G082300 | Chr03 | 10291132 | 10292046 | - | 3 | 199 | 22623.3 | 8.09 | SP |
| PdeMYB35 | Podel.03G100000 | Chr03 | 11903627 | 11905266 | - | 3 | 339 | 37856.1 | 7 | Nuclear |
| PdeMYB36 | Podel.03G120700 | Chr03 | 13675362 | 13677405 | + | 2 | 271 | 30646.2 | 5.1 | Nuclear |
| PdeMYB37 | Podel.03G141000 | Chr03 | 15310612 | 15312963 | - | 4 | 312 | 35035.9 | 5.73 | Nuclear |
| PdeMYB38 | Podel.03G156600 | Chr03 | 16561380 | 16562806 | - | 2 | 282 | 31315.1 | 9.31 | Nuclear |
| PdeMYB39 | Podel.03G156700 | Chr03 | 16567999 | 16569311 | + | 2 | 273 | 30799.3 | 9.23 | Nuclear |
| PdeMYB40 | Podel.03G167300 | Chr03 | 17247921 | 17249097 | + | 2 | 341 | 38339.1 | 7.48 | Nuclear |
| PdeMYB41 | Podel.03G207200 | Chr03 | 20347987 | 20353470 | + | 3 | 569 | 62236.4 | 4.81 | Nuclear |
| PdeMYB42 | Podel.03G240000 | Chr03 | 22612912 | 22614928 | - | 2 | 330 | 36179 | 8.32 | SP |
| PdeMYB43 | Podel.04G026900 | Chr04 | 2066121 | 2067846 | - | 3 | 255 | 28886.8 | 8.71 | SP |
| PdeMYB44 | Podel.04G033300 | Chr04 | 2639284 | 2640789 | - | 3 | 377 | 42374.7 | 5.91 | Nuclear |
| PdeMYB45 | Podel.04G093700 | Chr04 | 8361814 | 8363555 | - | 3 | 203 | 23424.1 | 10.22 | Nuclear |
| PdeMYB46 | Podel.04G095200 | Chr04 | 8525775 | 8528225 | + | 3 | 246 | 27186.3 | 9.31 | SP |
| PdeMYB47 | Podel.04G124200 | Chr04 | 12118859 | 12120127 | + | 3 | 215 | 25084.4 | 8.45 | Nuclear |
| PdeMYB48 | Podel.04G126400 | Chr04 | 12494275 | 12495336 | + | 3 | 213 | 24805 | 8.45 | Nuclear |
| PdeMYB49 | Podel.04G143500 | Chr04 | 15703135 | 15704594 | - | 3 | 319 | 35809.9 | 8.41 | Nuclear |
| PdeMYB50 | Podel.04G176700 | Chr04 | 18781390 | 18782694 | - | 2 | 269 | 29852.6 | 8.67 | Nuclear |
| PdeMYB51 | Podel.04G220900 | Chr04 | 22250498 | 22252361 | - | 3 | 341 | 38139.2 | 6.62 | Nuclear |
| PdeMYB52 | Podel.04G237800 | Chr04 | 23731529 | 23733114 | - | 3 | 309 | 35718.5 | 4.87 | SP |
| PdeMYB53 | Podel.04G237900 | Chr04 | 23738291 | 23740050 | - | 3 | 299 | 34631.6 | 8.3 | Nuclear |
| PdeMYB54 | Podel.04G238000 | Chr04 | 23753649 | 23755117 | - | 3 | 299 | 33925.7 | 6.88 | Nuclear |
| PdeMYB55 | Podel.05G000500 | Chr05 | 32300 | 34389 | - | 3 | 442 | 49114.2 | 7.48 | Nuclear |
| PdeMYB56 | Podel.05G080900 | Chr05 | 5536971 | 5538452 | - | 3 | 342 | 37957.5 | 6.7 | Nuclear |
| PdeMYB57 | Podel.05G108000 | Chr05 | 7907850 | 7910987 | - | 3 | 338 | 38430.4 | 7.9 | Nuclear |
| PdeMYB58 | Podel.05G124900 | Chr05 | 9347563 | 9348382 | - | 3 | 233 | 26205.7 | 8.81 | Nuclear |
| PdeMYB59 | Podel.05G155600 | Chr05 | 13353691 | 13354722 | + | 1 | 344 | 37093.3 | 8.22 | SP |
| PdeMYB60 | Podel.05G174200 | Chr05 | 17320547 | 17322059 | - | 3 | 338 | 37859.1 | 5.85 | Nuclear |
| PdeMYB61 | Podel.05G238200 | Chr05 | 23993007 | 23995138 | + | 3 | 274 | 31158.6 | 5.24 | Nuclear |
| PdeMYB62 | Podel.06G071900 | Chr06 | 5443778 | 5445768 | - | 3 | 233 | 27044.3 | 8.99 | SP |
| PdeMYB63 | Podel.06G133500 | Chr06 | 10906751 | 10908673 | + | 3 | 216 | 23948.4 | 10.33 | Nuclear |
| PdeMYB64 | Podel.06G180300 | Chr06 | 17920136 | 17921969 | + | 3 | 366 | 41010.6 | 6.93 | Nuclear |
| PdeMYB65 | Podel.06G233700 | Chr06 | 23932139 | 23933625 | + | 4 | 199 | 22763.5 | 9.76 | Nuclear |
| PdeMYB66 | Podel.06G234000 | Chr06 | 23953403 | 23955120 | - | 3 | 268 | 29924.5 | 6.53 | Nuclear |
| PdeMYB67 | Podel.06G234300 | Chr06 | 23990051 | 23991463 | + | 3 | 253 | 28199.6 | 9.5 | Nuclear |
| PdeMYB68 | Podel.06G247000 | Chr06 | 24866908 | 24869500 | - | 3 | 303 | 32909.8 | 5.72 | Nuclear |
| PdeMYB69 | Podel.06G289100 | Chr06 | 27887670 | 27889293 | + | 3 | 304 | 34058.7 | 6 | Nuclear |
| PdeMYB70 | Podel.07G010100 | Chr07 | 815088 | 816700 | - | 3 | 345 | 38209.7 | 6.65 | Nuclear |
| PdeMYB71 | Podel.07G075700 | Chr07 | 8963623 | 8967214 | + | 3 | 370 | 41347.4 | 5.88 | Nuclear |
| PdeMYB72 | Podel.07G101400 | Chr07 | 12360950 | 12362927 | + | 3 | 342 | 37803.4 | 6.84 | Nuclear |
| PdeMYB73 | Podel.07G145100 | Chr07 | 15276934 | 15278908 | - | 3 | 248 | 28931.1 | 8.67 | Nuclear |
| PdeMYB74 | Podel.08G011600 | Chr08 | 780600 | 782253 | - | 3 | 319 | 35478.5 | 8.78 | Nuclear |
| PdeMYB75 | Podel.08G077300 | Chr08 | 5128128 | 5132551 | - | 2 | 413 | 44665.4 | 5.98 | SP |
| PdeMYB76 | Podel.08G086000 | Chr08 | 5693644 | 5694417 | + | 1 | 258 | 28115.4 | 8.51 | Nuclear |
| PdeMYB77 | Podel.08G096800 | Chr08 | 6441586 | 6443488 | - | 3 | 339 | 38067.6 | 6.91 | SP |
| PdeMYB78 | Podel.08G105600 | Chr08 | 7073637 | 7075423 | - | 3 | 443 | 48968.1 | 7.75 | SP |
| PdeMYB79 | Podel.08G107000 | Chr08 | 7184877 | 7186706 | + | 3 | 383 | 42079 | 8.22 | SP |
| PdeMYB80 | Podel.08G120900 | Chr08 | 8156923 | 8158962 | + | 3 | 331 | 37066.8 | 6.86 | Nuclear |
| PdeMYB81 | Podel.08G143100 | Chr08 | 9788622 | 9790411 | + | 3 | 315 | 36000 | 6.62 | Nuclear |
| PdeMYB82 | Podel.08G149100 | Chr08 | 10219627 | 10221334 | + | 3 | 222 | 24928 | 8.73 | Nuclear |
| PdeMYB83 | Podel.08G191100 | Chr08 | 13577083 | 13579418 | + | 3 | 288 | 32733.4 | 5.66 | Nuclear |
| PdeMYB84 | Podel.08G197300 | Chr08 | 14014993 | 14016479 | + | 3 | 264 | 30452.4 | 9.18 | Nuclear |
| PdeMYB85 | Podel.08G206300 | Chr08 | 14590375 | 14591533 | + | 3 | 116 | 13378.5 | 10.88 | Nuclear |
| PdeMYB86 | Podel.08G206800 | Chr08 | 14629827 | 14631670 | - | 3 | 193 | 21982.4 | 9.32 | Nuclear |
| PdeMYB87 | Podel.08G208800 | Chr08 | 14749976 | 14750951 | + | 4 | 223 | 25368.5 | 7.48 | Nuclear |
| PdeMYB88 | Podel.08G211200 | Chr08 | 15002040 | 15003882 | + | 3 | 169 | 19242.2 | 9.6 | SP |
| PdeMYB89 | Podel.09G004800 | Chr09 | 922092 | 924106 | + | 3 | 338 | 37950 | 6.98 | Nuclear |
| PdeMYB90 | Podel.09G026900 | Chr09 | 3687470 | 3688865 | - | 3 | 244 | 27448.5 | 6.77 | SP |
| PdeMYB91 | Podel.09G054800 | Chr09 | 5751605 | 5754385 | - | 2 | 333 | 37023.2 | 5.08 | Nuclear |
| PdeMYB92 | Podel.09G063300 | Chr09 | 6370577 | 6373022 | - | 2 | 333 | 37598.6 | 5.25 | Nuclear |
| PdeMYB93 | Podel.09G098700 | Chr09 | 8825171 | 8825904 | - | 2 | 235 | 25677.7 | 7.15 | SP |
| PdeMYB94 | Podel.09G139200 | Chr09 | 11133384 | 11134762 | - | 2 | 272 | 30115.9 | 8.35 | Nuclear |
| PdeMYB95 | Podel.10G004300 | Chr10 | 395588 | 397625 | + | 3 | 353 | 39955.4 | 8.04 | Nuclear |
| PdeMYB96 | Podel.10G058300 | Chr10 | 7793594 | 7795184 | - | 3 | 166 | 19090.4 | 10.95 | Nuclear |
| PdeMYB97 | Podel.10G112700 | Chr10 | 11997927 | 11999496 | - | 3 | 227 | 25587.7 | 9.35 | Nuclear |
| PdeMYB98 | Podel.10G122300 | Chr10 | 12724528 | 12726031 | - | 3 | 317 | 35807.8 | 6.26 | Nuclear |
| PdeMYB99 | Podel.10G142600 | Chr10 | 14082980 | 14085960 | - | 3 | 366 | 40733.3 | 8.27 | Nuclear |
| PdeMYB100 | Podel.10G151200 | Chr10 | 14641264 | 14643226 | - | 3 | 318 | 35948.6 | 6.18 | Nuclear |
| PdeMYB101 | Podel.10G167500 | Chr10 | 15818270 | 15819881 | - | 3 | 383 | 41632.2 | 6.93 | Nuclear |
| PdeMYB102 | Podel.10G169100 | Chr10 | 15955356 | 15957463 | + | 3 | 450 | 49867.9 | 6.98 | Nuclear |
| PdeMYB103 | Podel.10G176300 | Chr10 | 16419589 | 16421305 | + | 3 | 338 | 37681.2 | 7.4 | Nuclear |
| PdeMYB104 | Podel.10G197600 | Chr10 | 17819845 | 17824391 | + | 2 | 413 | 44480.1 | 5.87 | SP |
| PdeMYB105 | Podel.11G036200 | Chr11 | 3214684 | 3216292 | - | 3 | 376 | 42278.4 | 6.18 | Nuclear |
| PdeMYB106 | Podel.11G123000 | Chr11 | 14999501 | 15001333 | - | 3 | 364 | 41073.8 | 7.43 | SP |
| PdeMYB107 | Podel.11G164500 | Chr11 | 18142065 | 18143307 | + | 3 | 328 | 36203.5 | 7.22 | Nuclear |
| PdeMYB108 | Podel.12G041600 | Chr12 | 3395104 | 3396935 | + | 3 | 244 | 28774.7 | 7.36 | Nuclear |
| PdeMYB109 | Podel.12G057100 | Chr12 | 5435192 | 5437132 | - | 3 | 328 | 37278.7 | 7.94 | SP |
| PdeMYB110 | Podel.12G076100 | Chr12 | 9076008 | 9077820 | + | 3 | 378 | 42076.8 | 5.42 | Nuclear |
| PdeMYB111 | Podel.12G083500 | Chr12 | 9913370 | 9915318 | + | 3 | 217 | 24713.8 | 8.49 | Nuclear |
| PdeMYB112 | Podel.12G087800 | Chr12 | 10573298 | 10575263 | + | 3 | 371 | 41730.5 | 6.84 | Nuclear |
| PdeMYB113 | Podel.12G135000 | Chr12 | 14232884 | 14235426 | + | 2 | 277 | 30629.8 | 5.17 | Nuclear |
| PdeMYB114 | Podel.12G149100 | Chr12 | 15258324 | 15260867 | + | 3 | 294 | 33331.1 | 8.02 | SP |
| PdeMYB115 | Podel.12G149300 | Chr12 | 15268698 | 15272000 | + | 3 | 299 | 33592.8 | 6.26 | Nuclear |
| PdeMYB116 | Podel.13G002600 | Chr13 | 170422 | 172637 | - | 3 | 447 | 49479.7 | 6.88 | Nuclear |
| PdeMYB117 | Podel.13G059800 | Chr13 | 4402731 | 4404624 | - | 2 | 305 | 34327.9 | 7.5 | SP |
| PdeMYB118 | Podel.13G059900 | Chr13 | 4415406 | 4417393 | - | 2 | 304 | 34209 | 7.86 | SP |
| PdeMYB119 | Podel.13G071000 | Chr13 | 5513801 | 5516718 | - | 3 | 395 | 43990.6 | 8.23 | SP |
| PdeMYB120 | Podel.13G071500 | Chr13 | 5662672 | 5664266 | - | 3 | 324 | 36501.5 | 6.52 | Nuclear |
| PdeMYB121 | Podel.13G082500 | Chr13 | 7261738 | 7263641 | + | 1 | 330 | 35599.5 | 8.46 | SP |
| PdeMYB122 | Podel.13G116900 | Chr13 | 12618002 | 12619170 | + | 4 | 289 | 33068.8 | 9.04 | Nuclear |
| PdeMYB123 | Podel.13G185700 | Chr13 | 16274813 | 16276270 | + | 3 | 363 | 40360.6 | 5.05 | Nuclear |
| PdeMYB124 | Podel.13G186200 | Chr13 | 16323512 | 16324936 | - | 3 | 267 | 30451.2 | 4.69 | SP |
| PdeMYB125 | Podel.13G186300 | Chr13 | 16331620 | 16334127 | - | 3 | 257 | 29324.1 | 4.43 | Nuclear |
| PdeMYB126 | Podel.14G022100 | Chr14 | 1572783 | 1574133 | - | 1 | 312 | 33650.6 | 8.76 | Nuclear |
| PdeMYB127 | Podel.14G034700 | Chr14 | 2537568 | 2538311 | + | 1 | 248 | 27352 | 7.56 | Nuclear |
| PdeMYB128 | Podel.14G082700 | Chr14 | 6004838 | 6007421 | + | 3 | 317 | 34545.6 | 7.04 | Nuclear |
| PdeMYB129 | Podel.14G103500 | Chr14 | 7511621 | 7513743 | - | 2 | 270 | 30707.7 | 10.08 | Nuclear |
| PdeMYB130 | Podel.14G115000 | Chr14 | 8436381 | 8438521 | - | 3 | 465 | 51770.1 | 8.27 | Nuclear |
| PdeMYB131 | Podel.14G121300 | Chr14 | 8941102 | 8942613 | + | 3 | 288 | 32688.1 | 6.23 | Nuclear |
| PdeMYB132 | Podel.14G127200 | Chr14 | 9347042 | 9350314 | + | 3 | 418 | 46474.7 | 5.07 | Nuclear |
| PdeMYB133 | Podel.15G035000 | Chr15 | 3052463 | 3054608 | - | 3 | 237 | 28044.2 | 7.95 | Nuclear |
| PdeMYB134 | Podel.15G043300 | Chr15 | 4178182 | 4180603 | + | 3 | 192 | 21782.2 | 9.26 | SP |
| PdeMYB135 | Podel.15G049500 | Chr15 | 5039123 | 5041360 | - | 3 | 334 | 37815.3 | 6.72 | SP |
| PdeMYB136 | Podel.15G049600 | Chr15 | 5107639 | 5109242 | - | 5 | 296 | 33510.4 | 6.1 | Nuclear |
| PdeMYB137 | Podel.15G070800 | Chr15 | 9116968 | 9119410 | + | 3 | 386 | 42909.9 | 5.65 | Nuclear |
| PdeMYB138 | Podel.15G079200 | Chr15 | 10000645 | 10001934 | + | 3 | 213 | 24426.6 | 9.07 | Nuclear |
| PdeMYB139 | Podel.15G079300 | Chr15 | 10016998 | 10018217 | + | 3 | 217 | 24777.9 | 8.37 | Nuclear |
| PdeMYB140 | Podel.15G081400 | Chr15 | 10335068 | 10336930 | + | 3 | 453 | 51120.1 | 6.13 | SP |
| PdeMYB141 | Podel.15G087100 | Chr15 | 10818647 | 10820624 | + | 3 | 370 | 41734.2 | 6.65 | Nuclear |
| PdeMYB142 | Podel.15G131200 | Chr15 | 14089556 | 14092231 | + | 2 | 274 | 30721 | 6.1 | SP |
| PdeMYB143 | Podel.15G145700 | Chr15 | 14964223 | 14966772 | + | 3 | 294 | 33287.1 | 7.36 | SP |
| PdeMYB144 | Podel.15G145800 | Chr15 | 14971274 | 14973878 | + | 3 | 297 | 32876.9 | 7.02 | Nuclear |
| PdeMYB145 | Podel.16G103700 | Chr16 | 9815297 | 9816690 | + | 4 | 291 | 32177.8 | 8.21 | Nuclear |
| PdeMYB146 | Podel.17G018100 | Chr17 | 1675322 | 1676618 | + | 4 | 222 | 25662.6 | 8.77 | Nuclear |
| PdeMYB147 | Podel.17G076000 | Chr17 | 8007534 | 8010945 | + | 3 | 193 | 21955.3 | 6.52 | SP |
| PdeMYB148 | Podel.17G086000 | Chr17 | 9461929 | 9463673 | + | 3 | 323 | 35715.7 | 7.37 | SP |
| PdeMYB149 | Podel.17G088900 | Chr17 | 10034052 | 10036237 | - | 3 | 437 | 48645.2 | 8.25 | SP |
| PdeMYB150 | Podel.17G090200 | Chr17 | 10160927 | 10163281 | + | 3 | 459 | 50425.9 | 7.15 | Nuclear |
| PdeMYB151 | Podel.17G101100 | Chr17 | 11222570 | 11223685 | + | 3 | 208 | 24176.3 | 7.76 | Nuclear |
| PdeMYB152 | Podel.17G102100 | Chr17 | 11298221 | 11299348 | + | 3 | 181 | 21109.1 | 10.33 | Nuclear |
| PdeMYB153 | Podel.17G134900 | Chr17 | 13853344 | 13858398 | + | 3 | 269 | 30684.3 | 7.24 | Nuclear |
| PdeMYB154 | Podel.17G135000 | Chr17 | 13861793 | 13863310 | + | 3 | 272 | 30715 | 9.52 | Nuclear |
| PdeMYB155 | Podel.17G135100 | Chr17 | 13890588 | 13892772 | + | 3 | 274 | 30961.8 | 5.54 | Nuclear |
| PdeMYB156 | Podel.17G135200 | Chr17 | 13910565 | 13912448 | + | 3 | 259 | 29334.3 | 8.35 | Nuclear |
| PdeMYB157 | Podel.17G135300 | Chr17 | 13925213 | 13927052 | + | 3 | 228 | 25831.6 | 10.64 | Nuclear |
| PdeMYB158 | Podel.17G137600 | Chr17 | 14104528 | 14106096 | - | 3 | 245 | 27125.4 | 8.93 | SP |
| PdeMYB159 | Podel.17G138900 | Chr17 | 14207097 | 14209296 | + | 3 | 257 | 29257.3 | 6.25 | Nuclear |
| PdeMYB160 | Podel.18G006000 | Chr18 | 492432 | 494045 | - | 3 | 424 | 47231.8 | 6.07 | SP |
| PdeMYB161 | Podel.18G006200 | Chr18 | 524903 | 526514 | - | 3 | 424 | 47231.8 | 6.07 | SP |
| PdeMYB162 | Podel.18G048700 | Chr18 | 4607196 | 4608297 | + | 3 | 201 | 23257.3 | 9.74 | Nuclear |
| PdeMYB163 | Podel.18G048900 | Chr18 | 4633546 | 4634678 | - | 3 | 297 | 33406.1 | 7.74 | Nuclear |
| PdeMYB164 | Podel.18G049300 | Chr18 | 4722395 | 4723360 | + | 3 | 209 | 24085.4 | 10.09 | Nuclear |
| PdeMYB165 | Podel.18G049500 | Chr18 | 4749177 | 4749924 | - | 3 | 138 | 15731.9 | 10.38 | Nuclear |
| PdeMYB166 | Podel.18G057900 | Chr18 | 6132278 | 6134358 | + | 3 | 307 | 33537.3 | 5.41 | Nuclear |
| PdeMYB167 | Podel.18G096800 | Chr18 | 12388374 | 12390199 | + | 3 | 367 | 41057.5 | 5.78 | Nuclear |
| PdeMYB168 | Podel.18G129100 | Chr18 | 14794104 | 14795161 | - | 3 | 232 | 26421.4 | 8.19 | Nuclear |
| PdeMYB169 | Podel.18G135800 | Chr18 | 15564690 | 15565916 | - | 3 | 231 | 26293.2 | 8.19 | Nuclear |
| PdeMYB170 | Podel.19G019100 | Chr19 | 1972272 | 1974060 | + | 3 | 219 | 25075.2 | 9.49 | Nuclear |
| PdeMYB171 | Podel.19G036400 | Chr19 | 3839709 | 3841692 | + | 2 | 176 | 19968.9 | 10.82 | SP |
| PdeMYB172 | Podel.19G042700 | Chr19 | 4770589 | 4773499 | - | 3 | 382 | 42599.4 | 8.79 | SP |
| PdeMYB173 | Podel.19G047500 | Chr19 | 5794800 | 5796746 | - | 3 | 326 | 37057.9 | 6.32 | Nuclear |
| PdeMYB174 | Podel.19G052400 | Chr19 | 7030377 | 7032216 | + | 3 | 341 | 38233.5 | 6.35 | SP |
| PdeMYB175 | Podel.19G084600 | Chr19 | 11366151 | 11367525 | + | 3 | 299 | 33817.6 | 8.94 | SP |
| PdeMYB176 | Podel.19G119700 | Chr19 | 14286918 | 14288685 | + | 3 | 365 | 40682.3 | 5.91 | Nuclear |
| PdeMYB177 | Podel.19G120200 | Chr19 | 14337201 | 14338678 | - | 3 | 265 | 30420.3 | 4.69 | Nuclear |
| PdeMYB178 | Podel.19G120300 | Chr19 | 14340796 | 14342165 | - | 3 | 264 | 30264.5 | 4.98 | Nuclear |
| PdeMYB179 | Podel.19G120400 | Chr19 | 14352939 | 14355042 | - | 3 | 251 | 28444.3 | 4.48 | Nuclear |
| PdeMYB180 | Podel.T101800 | scaffold_237 | 38096 | 39705 | + | 3 | 383 | 41628.2 | 6.78 | Nuclear |
| PdeMYB181 | Podel.T165800 | scaffold_421 | 3199 | 4879 | - | 4 | 300 | 33630.1 | 10.25 | SP |
| PdeMYB182 | Podel.T229100 | scaffold_652 | 18370 | 19903 | + | 4 | 317 | 35142.1 | 7.91 | Nuclear |
| PdeMYB183 | Podel.T263700 | scaffold_784 | 9707 | 11183 | + | 4 | 283 | 31721.2 | 6.42 | Nuclear |
| PdeMYB184 | Podel.03G132400 | Chr03 | 14679902 | 14685155 | - | 6 | 407 | 45707.7 | 8.99 | Nuclear |
| PdeMYB185 | Podel.06G092400 | Chr06 | 7093184 | 7100488 | + | 8 | 550 | 60780.7 | 7.2 | Nuclear |
| PdeMYB186 | Podel.06G255000 | Chr06 | 25487703 | 25491023 | - | 7 | 320 | 35998.3 | 8.02 | SP |
| PdeMYB187 | Podel.14G080700 | Chr14 | 5887204 | 5890672 | - | 8 | 537 | 60163.7 | 9.2 | Nuclear |
| PdeMYB188 | Podel.18G046700 | Chr18 | 4326220 | 4332114 | + | 8 | 717 | 79574.3 | 5.45 | SP |
| PdeMYB189 | Podel.15G043700 | Chr15 | 4230996 | 4240302 | + | 9 | 1078 | 120572.5 | 9.27 | Nuclear |
| PdeMYB190 | Podel.01G005600 | Chr01 | 358984 | 359314 | + | 1 | 51 | 5608.4 | 8.77 | SP |
| PdeMYB191 | Podel.01G083500 | Chr01 | 6633103 | 6635414 | + | 6 | 345 | 37031.8 | 10.35 | Nuclear |
| PdeMYB192 | Podel.01G166800 | Chr01 | 13882957 | 13885802 | + | 3 | 338 | 36311.8 | 7.36 | Nuclear |
| PdeMYB193 | Podel.01G200100 | Chr01 | 17866666 | 17870660 | + | 3 | 372 | 40310.6 | 7.19 | Nuclear |
| PdeMYB194 | Podel.02G116500 | Chr02 | 8596657 | 8601455 | - | 6 | 683 | 72025.8 | 6.8 | SP |
| PdeMYB195 | Podel.02G180900 | Chr02 | 13770048 | 13774481 | - | 8 | 337 | 38006.8 | 8.05 | Nuclear |
| PdeMYB196 | Podel.02G182300 | Chr02 | 13933278 | 13938432 | - | 6 | 296 | 32357.8 | 8.57 | Nuclear |
| PdeMYB197 | Podel.02G196300 | Chr02 | 15325682 | 15327326 | + | 2 | 300 | 33237.5 | 9.35 | SP |
| PdeMYB198 | Podel.02G199800 | Chr02 | 15862231 | 15870906 | + | 5 | 620 | 66819.5 | 5.95 | SP |
| PdeMYB199 | Podel.03G051700 | Chr03 | 6725371 | 6733631 | - | 3 | 379 | 40766.2 | 7.78 | Nuclear |
| PdeMYB200 | Podel.03G082700 | Chr03 | 10317173 | 10319845 | - | 3 | 338 | 36340.7 | 7.39 | Nuclear |
| PdeMYB201 | Podel.04G079800 | Chr04 | 7183929 | 7187982 | + | 8 | 318 | 34499.4 | 9 | SP |
| PdeMYB202 | Podel.04G080700 | Chr04 | 7273162 | 7275593 | - | 6 | 493 | 54387.2 | 6.46 | Nuclear |
| PdeMYB203 | Podel.06G145800 | Chr06 | 12228084 | 12232503 | + | 8 | 279 | 30509.3 | 8.71 | SP |
| PdeMYB204 | Podel.06G163300 | Chr06 | 14452186 | 14453429 | + | 3 | 304 | 33169 | 9.75 | Nuclear |
| PdeMYB205 | Podel.06G243000 | Chr06 | 24616143 | 24617575 | + | 3 | 192 | 20730.1 | 9.57 | Nuclear |
| PdeMYB206 | Podel.08G221200 | Chr08 | 16039172 | 16041062 | - | 3 | 315 | 34431.1 | 9.22 | Nuclear |
| PdeMYB207 | Podel.10G032200 | Chr10 | 5452403 | 5454413 | + | 3 | 310 | 33785.6 | 9.3 | Nuclear |
| PdeMYB208 | Podel.12G040400 | Chr12 | 3279417 | 3283081 | - | 6 | 488 | 53757.3 | 6.45 | Nuclear |
| PdeMYB209 | Podel.12G077300 | Chr12 | 9171814 | 9174040 | - | 3 | 313 | 34025.7 | 9.53 | Nuclear |
| PdeMYB210 | Podel.14G091100 | Chr14 | 6571122 | 6575380 | - | 9 | 344 | 38140.4 | 8.73 | Nuclear |
| PdeMYB211 | Podel.14G111000 | Chr14 | 8168067 | 8175881 | + | 7 | 736 | 80015.2 | 6.64 | Nuclear |
| PdeMYB212 | Podel.15G032200 | Chr15 | 2717652 | 2721228 | - | 6 | 432 | 47867.5 | 7.44 | Nuclear |
| PdeMYB213 | Podel.15G071800 | Chr15 | 9227512 | 9229679 | - | 3 | 319 | 35147 | 10.37 | Nuclear |
| PdeMYB214 | Podel.15G072700 | Chr15 | 9306418 | 9308593 | - | 3 | 319 | 35175.1 | 10.37 | Nuclear |
| PdeMYB215 | Podel.16G087300 | Chr16 | 6780239 | 6783756 | - | 8 | 333 | 36020.5 | 8.85 | SP |
| PdeMYB216 | Podel.17G158900 | Chr17 | 15773599 | 15777020 | + | 6 | 482 | 53460.1 | 6.57 | Nuclear |
| PdeMYB217 | Podel.17G161300 | Chr17 | 15911237 | 15914984 | - | 8 | 314 | 34067.2 | 9.06 | SP |
| PdeMYB218 | Podel.T167200 | scaffold_428 | 32965 | 35504 | + | 6 | 209 | 24034.1 | 10.94 | SP |
| PdeMYB219 | Podel.T172700 | scaffold_448 | 20162 | 22706 | - | 9 | 403 | 44465.2 | 6.71 | Nuclear |
| PdeMYB220 | Podel.02G134400 | Chr02 | 10088389 | 10089291 | + | 4 | 264 | 29002.2 | 7.9 | Nuclear |
| PdeMYB221 | Podel.02G188300 | Chr02 | 14450659 | 14452349 | - | 4 | 114 | 13457.3 | 8.46 | SP |
| PdeMYB222 | Podel.04G013300 | Chr04 | 903149 | 903574 | + | 3 | 78 | 8756.6 | 7.36 | Nuclear |
| PdeMYB223 | Podel.04G021100 | Chr04 | 1528728 | 1529923 | - | 3 | 75 | 8532.1 | 4.08 | Nuclear |
| PdeMYB224 | Podel.05G120400 | Chr05 | 8937157 | 8937757 | - | 3 | 133 | 14851.8 | 8.98 | Nuclear |
| PdeMYB225 | Podel.06G131900 | Chr06 | 10788385 | 10789364 | - | 3 | 193 | 22434.6 | 5.95 | Nuclear |
| PdeMYB226 | Podel.07G133000 | Chr07 | 14505268 | 14506958 | + | 3 | 98 | 11232.2 | 4.21 | SP |
| PdeMYB227 | Podel.10G189200 | Chr10 | 17247404 | 17248222 | - | 4 | 115 | 12721.4 | 8.78 | SP |
| PdeMYB228 | Podel.11G020900 | Chr11 | 1644585 | 1645529 | - | 3 | 75 | 8596.3 | 4.14 | Nuclear |
| PdeMYB229 | Podel.12G023600 | Chr12 | 2006634 | 2007296 | - | 1 | 221 | 24676.7 | 4.48 | Nuclear |
| PdeMYB230 | Podel.12G033300 | Chr12 | 2691175 | 2693336 | - | 4 | 122 | 14866.9 | 10.3 | Nuclear |
| PdeMYB231 | Podel.14G097900 | Chr14 | 7076162 | 7079358 | - | 3 | 101 | 11824.4 | 8.68 | SP |
| PdeMYB232 | Podel.15G022800 | Chr15 | 1837956 | 1839372 | + | 3 | 96 | 11516.9 | 9.05 | Nuclear |
| PdeMYB233 | Podel.17G037900 | Chr17 | 3217364 | 3218566 | - | 3 | 75 | 8561.1 | 4.09 | Nuclear |
| PdeMYB234 | Podel.19G036300 | Chr19 | 3817758 | 3819406 | + | 4 | 289 | 32081.8 | 9.42 | SP |
| PdeMYB235 | Podel.T182700 | scaffold_488 | 11837 | 13048 | + | 5 | 224 | 24838.8 | 6.95 | Nuclear |
| PdeMYB236 | Podel.T259500 | scaffold_761 | 1578 | 2485 | + | 3 | 156 | 18015.2 | 9.86 | SP |
| PdeMYB237 | Podel.01G181000 | Chr01 | 15350341 | 15357692 | + | 8 | 606 | 66624 | 9.06 | Nuclear |
| PdeMYB238 | Podel.01G310800 | Chr01 | 32060138 | 32064213 | + | 6 | 297 | 32741.4 | 10.3 | SP |
| PdeMYB239 | Podel.03G066600 | Chr03 | 8790142 | 8798634 | - | 9 | 649 | 71231.2 | 8.74 | Nuclear |
| PdeMYB240 | Podel.06G056100 | Chr06 | 4142605 | 4143384 | + | 2 | 70 | 8194.5 | 10.43 | Nuclear |
| PdeMYB241 | Podel.06G160600 | Chr06 | 14059473 | 14062484 | + | 6 | 273 | 29827.2 | 8.61 | SP |
| PdeMYB242 | Podel.07G006800 | Chr07 | 550984 | 557086 | - | 6 | 322 | 34804 | 10.59 | Nuclear |
| PdeMYB243 | Podel.09G089700 | Chr09 | 8295175 | 8299635 | + | 7 | 318 | 35111.1 | 9.83 | SP |
| PdeMYB244 | Podel.14G007300 | Chr14 | 563016 | 567403 | + | 7 | 302 | 32850.8 | 10.57 | SP |
| PdeMYB245 | Podel.18G065400 | Chr18 | 8165629 | 8169140 | - | 7 | 280 | 30665.3 | 9.78 | SP |
| PdeMYB246 | Podel.T127300 | scaffold_307 | 8761 | 12940 | - | 6 | 297 | 32741.4 | 10.3 | SP |
| PdeMYB247 | Podel.01G015200 | Chr01 | 1011522 | 1017056 | + | 3 | 676 | 76088 | 10.26 | SP |
| PdeMYB248 | Podel.01G039600 | Chr01 | 2755476 | 2759366 | + | 1 | 648 | 73635.3 | 9.09 | SP |
| PdeMYB249 | Podel.01G239800 | Chr01 | 24885497 | 24890136 | - | 4 | 649 | 71972.8 | 9.44 | SP |
| PdeMYB250 | Podel.03G231400 | Chr03 | 22070410 | 22076893 | - | 3 | 678 | 76124.4 | 9.88 | SP |
| PdeMYB251 | Podel.04G038300 | Chr04 | 3051075 | 3055568 | - | 5 | 507 | 56328.6 | 10.54 | Nuclear |
| PdeMYB252 | Podel.09G022100 | Chr09 | 3233682 | 3237039 | - | 4 | 643 | 71362.5 | 10.06 | SP |
| PdeMYB253 | Podel.13G194800 | Chr13 | 16979464 | 16982340 | - | 4 | 260 | 28758.7 | 9.64 | Nuclear |
| PdeMYB254 | Podel.05G034300 | Chr05 | 2408367 | 2420655 | - | 17 | 1087 | 120826.4 | 9.71 | Nuclear |
| PdeMYB255 | Podel.08G245400 | Chr08 | 20150903 | 20162610 | - | 19 | 1161 | 129073.6 | 8.15 | Nuclear |
| PdeMYB256 | Podel.10G018300 | Chr10 | 3126551 | 3145599 | - | 19 | 1154 | 128182 | 7.97 | Nuclear |
| PdeMYB257 | Podel.13G020800 | Chr13 | 1284571 | 1296390 | - | 17 | 1085 | 121257.9 | 9.84 | Nuclear |
| PdeMYB258 | Podel.06G135200 | Chr06 | 11094982 | 11100768 | + | 14 | 785 | 87147.5 | 7.07 | SP |
| PdeMYB259 | Podel.16G095700 | Chr16 | 7912307 | 7918226 | - | 13 | 788 | 87441.8 | 6.89 | Nuclear |
| PdeMYB260 | Podel.02G037200 | Chr02 | 2517128 | 2519115 | + | 2 | 100 | 11147.4 | 9.69 | SP |
| PdeMYB261 | Podel.02G087800 | Chr02 | 6268196 | 6274414 | - | 9 | 802 | 88346.8 | 6.23 | Nuclear |
| PdeMYB262 | Podel.04G159300 | Chr04 | 17409509 | 17411015 | + | 2 | 89 | 9773.7 | 9.63 | Nuclear |
| PdeMYB263 | Podel.04G159900 | Chr04 | 17469487 | 17471124 | - | 2 | 101 | 11525.8 | 9.84 | SP |
| PdeMYB264 | Podel.05G136000 | Chr05 | 10290522 | 10292680 | + | 1 | 86 | 9613.5 | 9.12 | SP |
| PdeMYB265 | Podel.05G192100 | Chr05 | 19877540 | 19884203 | + | 9 | 797 | 87450.8 | 5.82 | Nuclear |
| PdeMYB266 | Podel.05G242200 | Chr05 | 24271154 | 24272978 | - | 1 | 85 | 9343.3 | 7.66 | SP |
| PdeMYB267 | Podel.07G028800 | Chr07 | 2276150 | 2277566 | + | 1 | 79 | 8864.8 | 9.55 | SP |
| PdeMYB268 | Podel.09G121500 | Chr09 | 10141045 | 10142580 | + | 2 | 97 | 10737.9 | 5.72 | SP |
| PdeMYB269 | Podel.09G122100 | Chr09 | 10180241 | 10182449 | - | 2 | 131 | 14721.2 | 6.01 | SP |
| PdeMYB270 | Podel.04G157700 | Chr04 | 17214695 | 17220296 | + | 7 | 1006 | 109352.5 | 4.6 | Nuclear |
| PdeMYB271 | Podel.09G119900 | Chr09 | 10024732 | 10030524 | + | 7 | 1011 | 109910.1 | 4.71 | Nuclear |
| PdeMYB272 | Podel.02G005300 | Chr02 | 308634 | 313269 | - | 5 | 476 | 53102.6 | 5.32 | Nuclear |
| PdeMYB273 | Podel.02G004500 | Chr02 | 273376 | 277019 | + | 6 | 502 | 55159.2 | 5.31 | SP |
| PdeMYB274 | Podel.15G105300 | Chr15 | 12104700 | 12108151 | - | 8 | 544 | 60009.2 | 4.71 | SP |
| PdeMYB275 | Podel.15G105900 | Chr15 | 12150805 | 12154520 | + | 7 | 573 | 63203 | 4.8 | SP |
| PdeMYB276 | Podel.14G175500 | Chr14 | 13470475 | 13475612 | + | 13 | 581 | 65354.3 | 6.25 | Nuclear |
| PdeMYB277 | Podel.16G008000 | Chr16 | 378711 | 383860 | - | 13 | 560 | 63048.4 | 6.76 | Nuclear |
| PdeMYB278 | Podel.06G019200 | Chr06 | 1323083 | 1328445 | + | 13 | 561 | 62949.3 | 6.63 | Nuclear |
| PdeMYB279 | Podel.01G229400 | Chr01 | 23319498 | 23320822 | + | 2 | 201 | 23415.2 | 9.98 | SP |
| PdeMYB280 | Podel.01G260200 | Chr01 | 26782080 | 26784921 | + | 2 | 198 | 22056 | 9.84 | Nuclear |
| PdeMYB281 | Podel.05G098000 | Chr05 | 6989072 | 6994231 | + | 2 | 296 | 31453.9 | 7.18 | Nuclear |
| PdeMYB282 | Podel.06G106200 | Chr06 | 8461042 | 8463510 | + | 2 | 308 | 34686.5 | 7.6 | Nuclear |
| PdeMYB283 | Podel.07G072800 | Chr07 | 8436215 | 8437343 | + | 2 | 241 | 26638.2 | 9.08 | SP |
| PdeMYB284 | Podel.07G085900 | Chr07 | 10489517 | 10493919 | - | 2 | 297 | 31534 | 7.24 | Nuclear |
| PdeMYB285 | Podel.08G022200 | Chr08 | 1369690 | 1371033 | - | 2 | 222 | 25065.6 | 6.68 | SP |
| PdeMYB286 | Podel.08G078900 | Chr08 | 5216363 | 5218799 | + | 2 | 293 | 33039 | 7.29 | Nuclear |
| PdeMYB287 | Podel.09G042000 | Chr09 | 4777338 | 4779925 | + | 2 | 309 | 34624.5 | 9.74 | SP |
| PdeMYB288 | Podel.10G195800 | Chr10 | 17729447 | 17732047 | - | 2 | 294 | 33593.5 | 6.61 | Nuclear |
| PdeMYB289 | Podel.10G225100 | Chr10 | 19643604 | 19651364 | - | 8 | 1717 | 188450.9 | 5.88 | Nuclear |
| PdeMYB290 | Podel.10G247900 | Chr10 | 21070542 | 21072161 | + | 2 | 235 | 26360.2 | 7.27 | SP |
| PdeMYB291 | Podel.12G062000 | Chr12 | 6171865 | 6173515 | + | 2 | 296 | 32188.2 | 7.58 | Nuclear |
| PdeMYB292 | Podel.16G118100 | Chr16 | 11766830 | 11769645 | + | 2 | 308 | 34530.5 | 9.33 | Nuclear |
| PdeMYB293 | Podel.T191300 | scaffold_515 | 14403 | 15934 | - | 2 | 222 | 25083.6 | 6.95 | SP |
| PdeMYB294 | Podel.01G158200 | Chr01 | 12988817 | 12990153 | - | 1 | 315 | 35095.9 | 9.92 | Nuclear |
| PdeMYB295 | Podel.03G207400 | Chr03 | 20364328 | 20368269 | - | 1 | 648 | 73574 | 8.56 | SP |
| PdeMYB296 | Podel.05G132000 | Chr05 | 9940258 | 9941734 | + | 3 | 275 | 31945.8 | 6 | Nuclear |
| PdeMYB297 | Podel.13G139900 | Chr13 | 14695470 | 14698000 | - | 2 | 675 | 73709.8 | 6.46 | Nuclear |
| PdeMYB298 | Podel.15G041900 | Chr15 | 3973400 | 3975545 | + | 3 | 69 | 7917.4 | 10.98 | Nuclear |
| PdeMYB299 | Podel.17G079100 | Chr17 | 8421081 | 8422430 | - | 3 | 330 | 37328.4 | 6.04 | SP |
| PdeMYB300 | Podel.T187400 | scaffold_50 | 94404 | 95878 | + | 5 | 323 | 35436.5 | 8.05 | Nuclear |
| PdeMYB301 | Podel.T257400 | scaffold_751 | 15121 | 21124 | + | 17 | 834 | 91350.5 | 5.02 | SP |
| PdeMYB302 | Podel.T259600 | scaffold_761 | 4184 | 5063 | - | 3 | 169 | 19588.9 | 9.75 | Nuclear |
