# Supplementary material for: Genome-wide analysis and expression profiles of PdeMYB transcription factors in colored-leaf poplar (Populus deltoids)
Source: BMC Plant Biol. 2021 Sep 23;21:432. doi: 10.1186/s12870-021-03212-1 (PMC8459500; doi:10.1186/s12870-021-03212-1)
Supplement: Supplementary file 2 — Additional file 2. Segmentally and tandemly duplicated PdeMYB gene pairs. [file 12870_2021_3212_MOESM2_ESM.docx]

**Additional file 2**. Segmentally and tandemly duplicated *PdeMYB* gene pairs.

| Duplicated gene1 | Duplicated gene2 | Ka | Ks | Ka/Ks | Estimated time (Mya) | Purifying selection | Type |
| --- | --- | --- | --- | --- | --- | --- | --- |
| PdeMYB293 | PdeMYB285 | 0.00599393 | 0.000120495 | 49.744 | 0.0040165 | no | WGD or segmental replication |
| PdeMYB236 | PdeMYB302 | 0.0035264 | 7.43E-05 | 47.4658 | 0.00247645 | no | Tandem |
| PdeMYB10 | PdeMYB11 | 0.00170187 | 3.79E-05 | 44.8716 | 0.001264253 | no | WGD or segmental replication |
| PdeMYB156 | PdeMYB157 | 0.0656037 | 0.0547025 | 1.19928 | 1.823416667 | no | Tandem |
| PdeMYB53 | PdeMYB54 | 0.130335 | 0.130349 | 0.999893 | 4.344966667 | yes | Tandem |
| PdeMYB208 | PdeMYB212 | 0.155229 | 0.301409 | 0.51501 | 10.04696667 | yes | WGD or segmental replication |
| PdeMYB155 | PdeMYB156 | 0.111796 | 0.220243 | 0.507602 | 7.341433333 | yes | Tandem |
| PdeMYB191 | PdeMYB283 | 0.570838 | 1.15305 | 0.495067 | 38.435 | yes | WGD or segmental replication |
| PdeMYB137 | PdeMYB110 | 0.0910352 | 0.189874 | 0.47945 | 6.329133333 | yes | WGD or segmental replication |
| PdeMYB276 | PdeMYB277 | 0.34832 | 0.745893 | 0.466983 | 24.8631 | yes | WGD or segmental replication |
| PdeMYB7 | PdeMYB33 | 0.137886 | 0.295402 | 0.466772 | 9.846733333 | yes | WGD or segmental replication |
| PdeMYB211 | PdeMYB198 | 0.0877211 | 0.201856 | 0.434572 | 6.728533333 | yes | WGD or segmental replication |
| PdeMYB114 | PdeMYB143 | 0.104144 | 0.242592 | 0.429295 | 8.0864 | yes | WGD or segmental replication |
| PdeMYB35 | PdeMYB6 | 0.083429 | 0.204263 | 0.408439 | 6.808766667 | yes | WGD or segmental replication |
| PdeMYB64 | PdeMYB167 | 0.0795848 | 0.195127 | 0.407861 | 6.504233333 | yes | WGD or segmental replication |
| PdeMYB119 | PdeMYB172 | 0.0995297 | 0.245348 | 0.405668 | 8.178266667 | yes | WGD or segmental replication |
| PdeMYB239 | PdeMYB237 | 0.0837901 | 0.210549 | 0.397959 | 7.0183 | yes | WGD or segmental replication |
| PdeMYB57 | PdeMYB71 | 0.103979 | 0.262761 | 0.395717 | 8.7587 | yes | WGD or segmental replication |
| PdeMYB12 | PdeMYB91 | 0.069203 | 0.174906 | 0.395659 | 5.8302 | yes | WGD or segmental replication |
| PdeMYB77 | PdeMYB103 | 0.105093 | 0.272631 | 0.385477 | 9.0877 | yes | WGD or segmental replication |
| PdeMYB2 | PdeMYB40 | 0.0935388 | 0.250542 | 0.373346 | 8.3514 | yes | WGD or segmental replication |
| PdeMYB166 | PdeMYB68 | 0.0869464 | 0.238073 | 0.365209 | 7.935766667 | yes | WGD or segmental replication |
| PdeMYB270 | PdeMYB271 | 0.0813544 | 0.227559 | 0.357509 | 7.5853 | yes | WGD or segmental replication |
| PdeMYB227 | PdeMYB76 | 0.156767 | 0.487573 | 0.321526 | 16.25243333 | yes | WGD or segmental replication |
| PdeMYB201 | PdeMYB217 | 0.0753968 | 0.240186 | 0.313911 | 8.0062 | yes | WGD or segmental replication |
| PdeMYB285 | PdeMYB290 | 0.236169 | 0.758789 | 0.311244 | 25.29296667 | yes | WGD or segmental replication |
| PdeMYB293 | PdeMYB290 | 0.235081 | 0.760098 | 0.309278 | 25.3366 | yes | WGD or segmental replication |
| PdeMYB9 | PdeMYB90 | 0.0787477 | 0.257243 | 0.306122 | 8.574766667 | yes | WGD or segmental replication |
| PdeMYB112 | PdeMYB141 | 0.0557374 | 0.194225 | 0.286974 | 6.474166667 | yes | WGD or segmental replication |
| PdeMYB259 | PdeMYB258 | 0.0729956 | 0.254611 | 0.286695 | 8.487033333 | yes | WGD or segmental replication |
| PdeMYB215 | PdeMYB203 | 0.066446 | 0.23422 | 0.283691 | 7.807333333 | yes | WGD or segmental replication |
| PdeMYB104 | PdeMYB75 | 0.0524886 | 0.186682 | 0.281166 | 6.222733333 | yes | WGD or segmental replication |
| PdeMYB278 | PdeMYB277 | 0.0617857 | 0.22332 | 0.276669 | 7.444 | yes | WGD or segmental replication |
| PdeMYB67 | PdeMYB162 | 0.420031 | 1.52832 | 0.274832 | 50.944 | yes | WGD or segmental replication |
| PdeMYB261 | PdeMYB265 | 0.0557425 | 0.205903 | 0.270721 | 6.863433333 | yes | WGD or segmental replication |
| PdeMYB151 | PdeMYB48 | 0.0701519 | 0.260712 | 0.269078 | 8.6904 | yes | WGD or segmental replication |
| PdeMYB151 | PdeMYB47 | 0.0706597 | 0.265136 | 0.266503 | 8.837866667 | yes | WGD or segmental replication |
| PdeMYB65 | PdeMYB162 | 0.0760588 | 0.296775 | 0.256284 | 9.8925 | yes | WGD or segmental replication |
| PdeMYB175 | PdeMYB122 | 0.0811493 | 0.318109 | 0.255099 | 10.60363333 | yes | WGD or segmental replication |
| PdeMYB192 | PdeMYB200 | 0.067953 | 0.272384 | 0.249476 | 9.079466667 | yes | WGD or segmental replication |
| PdeMYB210 | PdeMYB195 | 0.0854314 | 0.343694 | 0.248568 | 11.45646667 | yes | WGD or segmental replication |
| PdeMYB210 | PdeMYB196 | 0.0675549 | 0.279106 | 0.24204 | 9.303533333 | yes | WGD or segmental replication |
| PdeMYB241 | PdeMYB245 | 0.0604419 | 0.249855 | 0.241908 | 8.3285 | yes | WGD or segmental replication |
| PdeMYB152 | PdeMYB47 | 0.0677808 | 0.289469 | 0.234156 | 9.648966667 | yes | WGD or segmental replication |
| PdeMYB295 | PdeMYB248 | 0.0523232 | 0.224815 | 0.232739 | 7.493833333 | yes | WGD or segmental replication |
| PdeMYB152 | PdeMYB48 | 0.0638425 | 0.275359 | 0.231852 | 9.178633333 | yes | WGD or segmental replication |
| PdeMYB46 | PdeMYB158 | 0.0647647 | 0.285603 | 0.226765 | 9.5201 | yes | WGD or segmental replication |
| PdeMYB193 | PdeMYB199 | 0.0609203 | 0.293462 | 0.207592 | 9.782066667 | yes | WGD or segmental replication |
| PdeMYB96 | PdeMYB84 | 0.0464345 | 0.223834 | 0.20745 | 7.461133333 | yes | WGD or segmental replication |
| PdeMYB45 | PdeMYB159 | 0.049528 | 0.241436 | 0.205139 | 8.047866667 | yes | WGD or segmental replication |
| PdeMYB17 | PdeMYB147 | 0.0593487 | 0.290492 | 0.204304 | 9.683066667 | yes | WGD or segmental replication |
| PdeMYB233 | PdeMYB226 | 0.0324709 | 0.174067 | 0.186542 | 5.802233333 | yes | WGD or segmental replication |
| PdeMYB20 | PdeMYB107 | 0.0555954 | 0.303223 | 0.183348 | 10.10743333 | yes | WGD or segmental replication |
| PdeMYB70 | PdeMYB23 | 0.247804 | 1.39259 | 0.177945 | 46.41966667 | yes | WGD or segmental replication |
| PdeMYB284 | PdeMYB281 | 0.0433261 | 0.25661 | 0.168841 | 8.553666667 | yes | WGD or segmental replication |
| PdeMYB94 | PdeMYB50 | 0.0529808 | 0.327569 | 0.161739 | 10.91896667 | yes | WGD or segmental replication |
| PdeMYB133 | PdeMYB108 | 0.0617221 | 0.383663 | 0.160876 | 12.78876667 | yes | WGD or segmental replication |
| PdeMYB4 | PdeMYB37 | 0.0345959 | 0.217487 | 0.159071 | 7.249566667 | yes | WGD or segmental replication |
| PdeMYB15 | PdeMYB88 | 0.145968 | 0.932345 | 0.15656 | 31.07816667 | yes | WGD or segmental replication |
| PdeMYB223 | PdeMYB228 | 0.0229616 | 0.215861 | 0.106372 | 7.195366667 | yes | WGD or segmental replication |
| PdeMYB15 | PdeMYB85 | 0.110242 | 1.10116 | 0.100115 | 36.70533333 | yes | WGD or segmental replication |
| PdeMYB48 | PdeMYB47 | 0.0019248 | 0.0372826 | 0.0516273 | 1.242753333 | yes | WGD or segmental replication |
