# Supplementary material for: Genome-wide analysis and expression profiles of PdeMYB transcription factors in colored-leaf poplar (Populus deltoids)
Source: BMC Plant Biol. 2021 Sep 23;21:432. doi: 10.1186/s12870-021-03212-1 (PMC8459500; doi:10.1186/s12870-021-03212-1)
Supplement: Supplementary file 3 — Additional file 3. One-to-one orthologous relationships between Populus deltoids and Arabidopsis. [file 12870_2021_3212_MOESM3_ESM.docx]

**Additional file 3**. One-to-one orthologous relationships between *Populus deltoids* and *Arabidopsis*.

| *Populus deltoids* | Genomic Location | *Arabidopsis* | Genomic Location | E value |
| --- | --- | --- | --- | --- |
| PdeMYB57 | Chr05 | AT1G16490 | Chr01 | 9.00E-76 |
| PdeMYB150 | Chr17 | AT1G18570 | Chr01 | 8.00E-68 |
| PdeMYB150 | Chr17 | AT1G18710 | Chr01 | 6.00E-59 |
| PdeMYB78 | Chr8 | AT1G26780 | Chr01 | 7.00E-81 |
| PdeMYB100 | Chr10 | AT1G48000 | Chr01 | 1.00E-87 |
| PdeMYB243 | Chr9 | AT1G49950 | Chr01 | 6.00E-98 |
| PdeMYB57 | Chr5 | AT1G56160 | Chr01 | 5.00E-76 |
| PdeMYB154 | Chr17 | AT1G56650 | Chr02 | 1.00E-63 |
| PdeMYB159 | Chr17 | AT1G66230 | Chr02 | 8.00E-96 |
| PdeMYB154 | Chr17 | AT1G66380 | Chr02 | 5.00E-63 |
| PdeMYB154 | Chr17 | AT1G66390 | Chr03 | 9.00E-65 |
| PdeMYB206 | Chr08 | AT1G70000 | Chr03 | 4.00E-81 |
| PdeMYB180 | scaffold_237 | AT1G74080 | Chr03 | 2.00E-67 |
| PdeMYB150 | Chr17 | AT1G74430 | Chr03 | 5.00E-62 |
| PdeMYB57 | Chr05 | AT1G79180 | Chr03 | 4.00E-74 |
| PdeMYB185 | Chr06 | AT2G13960 | Chr03 | 1.00E-52 |
| PdeMYB248 | Chr01 | AT3G11450 | Chr03 | 0 |
| PdeMYB292 | Chr16 | AT2G38090 | Chr03 | 2.00E-128 |
| PdeMYB80 | Chr08 | AT2G47190 | Chr03 | 2.00E-77 |
| PdeMYB17 | Chr01 | AT3G01530 | Chr03 | 2.00E-78 |
| PdeMYB215 | Chr16 | AT3G09600 | Chr03 | 1.00E-129 |
| PdeMYB57 | Chr05 | AT3G12820 | Chr03 | 1.00E-67 |
| PdeMYB8 | Chr01 | AT3G13890 | Chr03 | 2.00E-81 |
| PdeMYB84 | Chr08 | AT3G24310 | Chr03 | 2.00E-96 |
| PdeMYB140 | Chr15 | AT3G27785 | Chr03 | 6.00E-57 |
| PdeMYB17 | Chr01 | AT3G27810 | Chr03 | 8.00E-83 |
| PdeMYB138 | Chr15 | AT3G27920 | Chr03 | 5.00E-61 |
| PdeMYB102 | Chr10 | AT3G29020 | Chr03 | 5.00E-67 |
| PdeMYB90 | Chr09 | AT3G53200 | Chr03 | 1.00E-50 |
| PdeMYB27 | Chr02 | AT3G61250 | Chr03 | 8.00E-135 |
| PdeMYB130 | Chr14 | AT4G01680 | Chr04 | 9.00E-94 |
| PdeMYB122 | Chr13 | AT4G09460 | Chr04 | 1.00E-93 |
| PdeMYB277 | Chr16 | AT4G16420 | Chr04 | 0 |
| PdeMYB5 | Chr01 | AT4G12350 | Chr04 | 3.00E-107 |
| PdeMYB176 | Chr19 | AT4G28110 | Chr04 | 1.00E-100 |
| PdeMYB133 | Chr15 | AT4G33450 | Chr04 | 1.00E-59 |
| PdeMYB70 | Chr07 | AT4G37780 | Chr04 | 4.00E-77 |
| PdeMYB50 | Chr04 | AT4G38620 | Chr04 | 6.00E-115 |
| PdeMYB292 | Chr16 | AT5G01200 | Chr05 | 9.00E-89 |
| PdeMYB215 | Chr16 | AT5G02840 | Chr05 | 7.00E-126 |
| PdeMYB290 | Chr10 | AT5G04760 | Chr05 | 1.00E-85 |
| PdeMYB150 | Chr17 | AT5G07690 | Chr05 | 5.00E-67 |
| PdeMYB101 | Chr10 | AT5G07700 | Chr05 | 2.00E-66 |
| PdeMYB281 | Chr05 | AT5G08520 | Chr05 | 3.00E-130 |
| PdeMYB159 | Chr17 | AT5G16600 | Chr05 | 7.00E-100 |
| PdeMYB119 | Chr13 | AT5G17800 | Chr05 | 3.00E-69 |
| PdeMYB89 | Chr09 | AT5G23000 | Chr05 | 8.00E-74 |
| PdeMYB116 | Chr13 | AT5G26660 | Chr05 | 3.00E-85 |
| PdeMYB67 | Chr06 | AT5G35550 | Chr05 | 3.00E-61 |
| PdeMYB248 | Chr01 | AT5G06110 | Chr05 | 0 |
| PdeMYB200 | Chr03 | AT5G47390 | Chr05 | 1.00E-143 |
| PdeMYB201 | Chr04 | AT5G52660 | Chr05 | 1.00E-132 |
| PdeMYB41 | Chr03 | AT5G55020 | Chr05 | 4.00E-67 |
| PdeMYB90 | Chr09 | AT5G59780 | Chr05 | 6.00E-68 |
| PdeMYB44 | Chr04 | AT5G60890 | Chr05 | 1.00E-67 |
| PdeMYB180 | scaffold_237 | AT5G61420 | Chr05 | 2.00E-68 |
| PdeMYB142 | Chr15 | AT5G62320 | Chr05 | 3.00E-72 |
| PdeMYB72 | Chr07 | AT5G65230 | Chr05 | 8.00E-103 |
| PdeMYB70 | Chr07 | AT5G65790 | Chr05 | 2.00E-83 |
| PdeMYB121 | Chr13 | AT5G67300 | Chr05 | 2.00E-111 |
