# Supplementary material for: Genome-wide analysis and expression profiles of PdeMYB transcription factors in colored-leaf poplar (Populus deltoids)
Source: BMC Plant Biol. 2021 Sep 23;21:432. doi: 10.1186/s12870-021-03212-1 (PMC8459500; doi:10.1186/s12870-021-03212-1)
Supplement: Supplementary file 4 — Additional file 4. One-to-one orthologous relationships between Populus deltoids and Oryza sativa. [file 12870_2021_3212_MOESM4_ESM.docx]

**Additional file 4**. One-to-one orthologous relationships between *Populus deltoids* and *Oryza sativa*.

| *Populus deltoids* | Genomic Location | *Oryza sativa* | Genomic Location | E value |
| --- | --- | --- | --- | --- |
| PdeMYB109 | Chr12 | LOC_Os01g03720 | Chr01 | 4.87E-84 |
| PdeMYB186 | Chr06 | LOC_Os01g12860 | Chr01 | 2.68E-98 |
| PdeMYB185 | Chr06 | LOC_Os01g12860 | Chr01 | 3.78E-81 |
| PdeMYB186 | Chr06 | LOC_Os01g62410 | Chr01 | 6.13E-93 |
| PdeMYB188 | Chr18 | LOC_Os01g62410 | Chr01 | 1.71E-89 |
| PdeMYB150 | Chr17 | LOC_Os02g36890 | Chr02 | 8.01E-106 |
| PdeMYB79 | Chr08 | LOC_Os02g36890 | Chr02 | 7.2E-102 |
| PdeMYB210 | Chr14 | LOC_Os02g45670 | Chr02 | 7.22E-112 |
| PdeMYB217 | Chr17 | LOC_Os02g45670 | Chr02 | 5.79E-109 |
| PdeMYB201 | Chr04 | LOC_Os02g45670 | Chr02 | 1.65E-108 |
| PdeMYB196 | Chr02 | LOC_Os02g45670 | Chr02 | 4.57E-92 |
| PdeMYB56 | Chr05 | LOC_Os02g51799 | Chr02 | 6.71E-101 |
| PdeMYB60 | Chr05 | LOC_Os02g51799 | Chr02 | 8.43E-100 |
| PdeMYB22 | Chr02 | LOC_Os02g51799 | Chr02 | 2.85E-95 |
| PdeMYB64 | Chr06 | LOC_Os02g54520 | Chr02 | 2.95E-87 |
| PdeMYB167 | Chr18 | LOC_Os02g54520 | Chr02 | 4.86E-85 |
| PdeMYB80 | Chr08 | LOC_Os03g20090 | Chr03 | 7.12E-95 |
| PdeMYB100 | Chr10 | LOC_Os03g20090 | Chr03 | 1.35E-92 |
| PdeMYB70 | Chr07 | LOC_Os03g56090 | Chr03 | 2E-83 |
| PdeMYB281 | Chr05 | LOC_Os04g58020 | Chr04 | 4.48E-122 |
| PdeMYB284 | Chr07 | LOC_Os04g58020 | Chr04 | 2.53E-121 |
| PdeMYB141 | Chr15 | LOC_Os05g04820 | Chr05 | 5.28E-83 |
| PdeMYB112 | Chr12 | LOC_Os05g04820 | Chr05 | 6.68E-83 |
| PdeMYB188 | Chr18 | LOC_Os05g38460 | Chr05 | 5.1E-77 |
| PdeMYB217 | Chr17 | LOC_Os06g01670 | Chr06 | 1.18E-104 |
| PdeMYB60 | Chr05 | LOC_Os06g02250 | Chr06 | 8.82E-89 |
| PdeMYB22 | Chr02 | LOC_Os06g02250 | Chr06 | 6.13E-86 |
| PdeMYB72 | Chr07 | LOC_Os06g02250 | Chr06 | 9.43E-86 |
| PdeMYB56 | Chr05 | LOC_Os06g02250 | Chr06 | 3.93E-85 |
| PdeMYB174 | Chr19 | LOC_Os06g02250 | Chr06 | 1.44E-84 |
| PdeMYB72 | Chr07 | LOC_Os06g11780 | Chr06 | 1.32E-105 |
| PdeMYB56 | Chr05 | LOC_Os06g11780 | Chr06 | 1.62E-105 |
| PdeMYB174 | Chr19 | LOC_Os06g11780 | Chr06 | 1.19E-83 |
| PdeMYB159 | Chr17 | LOC_Os06g14670 | Chr06 | 8.52E-89 |
| PdeMYB142 | Chr15 | LOC_Os06g14670 | Chr06 | 4.32E-88 |
| PdeMYB217 | Chr17 | LOC_Os06g45840 | Chr06 | 5.91E-111 |
| PdeMYB201 | Chr04 | LOC_Os06g45840 | Chr06 | 5.51E-109 |
| PdeMYB77 | Chr08 | LOC_Os07g31470 | Chr07 | 5.97E-87 |
| PdeMYB40 | Chr03 | LOC_Os07g31470 | Chr07 | 6.01E-83 |
| PdeMYB19 | Chr01 | LOC_Os07g37210 | Chr07 | 6.87E-93 |
| PdeMYB18 | Chr01 | LOC_Os07g37210 | Chr07 | 1.29E-90 |
| PdeMYB106 | Chr11 | LOC_Os07g37210 | Chr07 | 6.60E-87 |
| PdeMYB80 | Chr08 | LOC_Os07g48870 | Chr07 | 7.40E-91 |
| PdeMYB100 | Chr10 | LOC_Os07g48870 | Chr07 | 6.05E-90 |
| PdeMYB167 | Chr18 | LOC_Os08g15020 | Chr08 | 1.48E-89 |
| PdeMYB64 | Chr06 | LOC_Os08g15020 | Chr08 | 4.33E-88 |
| PdeMYB159 | Chr17 | LOC_Os08g33150 | Chr08 | 3.45E-99 |
| PdeMYB45 | Chr04 | LOC_Os08g33150 | Chr08 | 2.90E-90 |
| PdeMYB36 | Chr03 | LOC_Os08g33150 | Chr08 | 7.60E-85 |
| PdeMYB5 | Chr01 | LOC_Os08g33150 | Chr08 | 9.25E-85 |
| PdeMYB173 | Chr19 | LOC_Os08g33940 | Chr08 | 4.11E-86 |
| PdeMYB56 | Chr05 | LOC_Os08g37970 | Chr08 | 3.28E-105 |
| PdeMYB174 | Chr19 | LOC_Os08g37970 | Chr08 | 1.83E-83 |
| PdeMYB50 | Chr04 | LOC_Os08g43550 | Chr08 | 7.91E-107 |
| PdeMYB49 | Chr04 | LOC_Os08g43550 | Chr08 | 1.21E-87 |
| PdeMYB122 | Chr13 | LOC_Os08g43550 | Chr08 | 1.36E-87 |
| PdeMYB74 | Chr08 | LOC_Os08g43550 | Chr08 | 3.59E-87 |
| PdeMYB175 | Chr19 | LOC_Os08g43550 | Chr08 | 9.82E-87 |
| PdeMYB159 | Chr17 | LOC_Os09g23620 | Chr09 | 2.85E-94 |
| PdeMYB45 | Chr04 | LOC_Os09g23620 | Chr09 | 1.45E-90 |
| PdeMYB36 | Chr03 | LOC_Os09g23620 | Chr09 | 1.29E-86 |
| PdeMYB159 | Chr17 | LOC_Os09g36250 | Chr09 | 2.11E-87 |
| PdeMYB50 | Chr04 | LOC_Os09g36730 | Chr09 | 2.32E-115 |
| PdeMYB94 | Chr09 | LOC_Os09g36730 | Chr09 | 5.64E-115 |
| PdeMYB175 | Chr19 | LOC_Os09g36730 | Chr09 | 3.66E-91 |
| PdeMYB122 | Chr13 | LOC_Os09g36730 | Chr09 | 6.62E-90 |
| PdeMYB49 | Chr04 | LOC_Os09g36730 | Chr09 | 7.89E-89 |
| PdeMYB74 | Chr08 | LOC_Os09g36730 | Chr09 | 8.25E-89 |
| PdeMYB200 | Chr03 | LOC_Os10g41200 | Chr10 | 2.62E-86 |
| PdeMYB173 | Chr19 | LOC_Os11g35390 | Chr11 | 3.13E-85 |
| PdeMYB175 | Chr19 | LOC_Os12g07640 | Chr12 | 1.86E-89 |
| PdeMYB122 | Chr13 | LOC_Os12g07640 | Chr12 | 2.96E-89 |
| PdeMYB94 | Chr09 | LOC_Os12g07640 | Chr12 | 1.14E-88 |
| PdeMYB74 | Chr08 | LOC_Os12g07640 | Chr12 | 1.48E-87 |
| PdeMYB49 | Chr04 | LOC_Os12g07640 | Chr12 | 1.71E-87 |
| PdeMYB186 | Chr06 | LOC_Os12g13570 | Chr12 | 1.84E-117 |
| PdeMYB187 | Chr14 | LOC_Os12g13570 | Chr12 | 1.02E-81 |
