# Supplementary material for: Genome-wide analysis and expression profiles of PdeMYB transcription factors in colored-leaf poplar (Populus deltoids)
Source: BMC Plant Biol. 2021 Sep 23;21:432. doi: 10.1186/s12870-021-03212-1 (PMC8459500; doi:10.1186/s12870-021-03212-1)
Supplement: Supplementary file 5 — Additional file 5.The candidate PdeMYB genes with their expression level in the leaves of QHP more than ten times than these in L2025 or specifically expressed in the leaves of QHP or L2025. [file 12870_2021_3212_MOESM5_ESM.docx]

**Additional file 5**. The candidate *PdeMYB* genes with their expression level in the leaves of QHP more than ten times than these in L2025 or specifically expressed in the leaves of QHP or L2025.

| Gene name | Transcript abundance in L2025 | Transcript abundance in QHP | Function |
| --- | --- | --- | --- |
| PdeMYB4 | 37.062 | 1.578 | PREDICTED: protein ODORANT1-like |
| PdeMYB25 | 0 | 21.679 | transcription factor MYB73 |
| PdeMYB27 | 0 | 1.39 | transcription factor MYB41 |
| PdeMYB37 | 58.637 | 0 | transcription factor MYB46 isoform X1 |
| PdeMYB56 | 4.773 | 0 | transcription factor MYB93 |
| PdeMYB60 | 0 | 4.896 | transcription factor MYB93 isoform X1 |
| PdeMYB63 | 0 | 3.758 | transcription factor RAX2 |
| PdeMYB66 | 885.235 | 21.032 | transcription factor WER |
| PdeMYB70 | 0 | 9.096 | transcription factor RAX3 isoform X1 |
| PdeMYB72 | 0 | 1.827 | transcription factor MYB53 |
| PdeMYB96 | 0 | 2.271 | myb-related protein 305 |
| PdeMYB99 | 20.758 | 0 | transcription factor MYB111 |
| PdeMYB114 | 0 | 1.42 | hypothetical protein POPTR_012G140500v3 |
| PdeMYB143 | 0 | 4.25 | transcription factor LAF1 |
| PdeMYB154 | 1.165 | 15.473 | transcription factor MYB113 |
| PdeMYB155 | 1.618 | 53.737 | transcription factor MYB90 |
| PdeMYB160 | 0 | 1.119 | PREDICTED: myb-related protein 308-like isoform X3 |
| PdeMYB165 | 14.196 | 0 | transcription factor MYB8 |
| PdeMYB177 | 2.393 | 0 | transcription repressor MYB6 |
| PdeMYB179 | 52.105 | 5.151 | transcription factor MYB4 |
| PdeMYB181 | 4.208 | 0 | transcription repressor MYB5 |
| PdeMYB279 | 0 | 8.182 | transcription factor SRM1 |
| PdeMYB285 | 3.948 | 0 | hypothetical protein POPTR_008G016400v3, partial |
