# Supplementary material for: Genome-wide analysis and expression profiles of PdeMYB transcription factors in colored-leaf poplar (Populus deltoids)
Source: BMC Plant Biol. 2021 Sep 23;21:432. doi: 10.1186/s12870-021-03212-1 (PMC8459500; doi:10.1186/s12870-021-03212-1)
Supplement: Supplementary file 6 — Additional file 6. Specific primers used in relative quantitative real-time RT-PCR. [file 12870_2021_3212_MOESM6_ESM.docx]

**Additional file 6**. Specific primers used in relative quantitative real-time RT-PCR.

| **Gene name** | **Accession** | **Forward primer (5’to 3’)** | **Reverse primer (5’ to 3’)** |
| --- | --- | --- | --- |
| PdeMYB25 | Podel.02G141800 | CTTCAGATTCTTCCGCTTCG | TCGCTCAACAAGCCCAGT |
| PdeMYB27 | Podel.02G174500 | GTTTCTCAGGCATCTTCGT | TTTGATTGCTTGCTGTGGC |
| PdeMYB66 | Podel.06G234000 | GAAGAGGGAATAAAGACAC | TCATTTGAGTTGGGATTTG |
| PdeMYB99 | Podel.10G142600 | CTCGTGGAAATCACTACCT | TCATTTGAGTTGGGATTTG |
| PdeMYB155 | Podel.17G135100 | ACAGGTGGTCATTGATTGC | TTTCCAGCCTCTAACCAAC |
| PdeMYB179 | Podel.19G120400 | GGCGAGAACTCCTTGCTGT | GCAGCGATAGCAGACCAC |
| PdeMYB279 | Podel.01G229400 | CGAGGAAACGCCAAGACGG | TCCTTACTTCACGCCAACT |
| PdeMYB4 | Podel.01G103800 | CCTGGAAGAACAGACAACG | CGTACACTACTATCCGCAAT |
| PdeMYB37 | Podel.03G141000 | CTGGGCTTCAAAGATGTGG | ATCAACTTCTCCTCCTCTG |
| PdeMYB72 | Podel.07G101400 | TCAAGGATAGCCCAGTGTC | AAGGGATTTGTAAGTCAGG |
| PdeMYB143 | Podel.15G145700 | GGCTCAGAAACTATGTCCT | GAGACCACTTGTTGCCTAA |
| PdeMYB160 | Podel.18G006000 | TCAGAGGACAGAGAGGAGGC | CCATTTACCTTCACCATGG |
| PdeMYB165 | Podel.18G049500 | TGTTGCTCCAAGGAAGGACT | GAGTCTACAGCTCTTACCAC |
| PdeMYB177 | Podel.19G120200 | TGGACTCAGGAAAGGTGCAT | TAATCTGCAACTCTTGCCAC |
| PdeMYB285 | Podel.08G022200 | TCCAACTCGGTGTTTCACC | CGGTCTTTAACTTCCCACG |
| PdeMYB60 | Podel.05G174200 | CTGGAACACCCATTTGAAG | TCTAATGGACGATGCTCCA |
| PdeMYB70 | Podel.07G010100 | TGACAAAGCCAACGTGAAG | GACGGAGATAATTCAACCA |
| PdeMYB96 | Podel.10G058300 | TGGTCTCATTGAAGAGGGCT | CTCAATCTGCAGCTCTTTCC |
| PdeMYB114 | Podel.12G149100 | GTGGTCACCTGAAGAAGAT | CATCTTAATCTGCAGCTC |
| PdeMYB56 | Podel.05G080900 | TCTCCTTGTTGTGACGAG | TTGTCCACCTTAACCTGC |
| PdeMYB154 | Podel.17G135000 | TGCATGGACCGAAGAGGAAG | GTCCTCTCTTGATATTCGGC |
| PdeMYB181 | Podel.T165800 | AGCAGGAGTGTACCTACAC | CAGAAGGCCTAAGAAGTCC |
